# A phosphate-targeted dinuclear Cu(II) complex combining major groove binding and oxidative DNA cleavage

**DOI:** 10.1093/nar/gky806

**Published:** 2018-09-17

**Authors:** Zara Molphy, Diego Montagner, Satish S Bhat, Creina Slator, Conor Long, Andrea Erxleben, Andrew Kellett

**Affiliations:** 1 School of Chemical Sciences and National Institute for Cellular Biotechnology, Dublin City University, Glasnevin, Dublin 9, Ireland; 2 Department of Chemistry, Maynooth University, Maynooth, Kildare, Ireland; 3 School of Chemistry, National University of Ireland Galway, Galway, Ireland; 4 Synthesis and Solid-State Pharmaceutical Centre, School of Chemistry, National University of Ireland Galway, Galway, Ireland; 5 Synthesis and Solid-State Pharmaceutical Centre, School of Chemical Sciences, Dublin City University, Glasnevin, Dublin 9, Ireland

## Abstract

Free radical generation is an inevitable consequence of aerobic existence and is implicated in a wide variety of pathological conditions including cancer, cardiovascular disease, ageing and neurodegenerative disorder. Free radicals can, however, be used to our advantage since their production is catalysed by synthetic inorganic molecules—termed artificial metallonucleases—that cut DNA strands by oxidative cleavage reactions. Here, we report the rational design and DNA binding interactions of a novel *di*-Cu^2+^ artificial metallonuclease [Cu_2_(*tetra*-(2-pyridyl)-NMe-naphthalene)Cl_4_] (Cu_2_TPNap). Cu_2_TPNap is a high-affinity binder of duplex DNA with an apparent binding constant (*K*_app_) of 10^7^ M(bp)^−1^. The agent binds non-intercalatively in the major groove causing condensation and G-C specific destabilization. Artificial metallonuclease activity occurs in the absence of exogenous reductant, is dependent on superoxide and hydrogen peroxide, and gives rise to single strand DNA breaks. Pre-associative molecular docking studies with the 8-mer d(GGGGCCCC)_2_, a model for poly[d(G-C)_2_], identified selective major groove incorporation of the complex with ancillary Cu^2+^-phosphate backbone binding. Molecular mechanics methods then showed the d(GGGGCCCC)_2_ adduct to relax about the complex and this interaction is supported by UV melting experiments where poly[d(G-C)_2_] is selectively destabilized.

## INTRODUCTION

Reactive oxygen species (ROS) play an important role in human health and disease progression. They are endogenously produced by oxygen metabolism in the mitochondria and by several exogenous factors including ionizing radiation, redox metal ion overload, and therapeutic drug administration. DNA is susceptible to oxidation at nucleobases and also deoxyribose groups, leading to direct strand breakages (1–3). Numerous oxidative DNA damage lesions are known and in order for the cell to avert the loss of genetic information, repair enzymes function to remove oxidative lesions to restore self-complementary base pairing (4). But since genome integrity is crucial for cell survival, small molecules, natural products, and ionizing radiation are applied as therapeutic oxidants to trigger DNA damage and cell death.

Metal complexes of iron and copper catalyse oxidative DNA damage and significant interest is focused on developing new DNA-targeted metallodrugs with these ions (5–9). Part of this interest stems from the clinical antitumoural antibiotic bleomycin, which is active against a range of lymphomas, germ-cell tumours and carcinomas of the head and neck (10). Bleomycin catalyses double strand breaks (DSBs) by damaging C-4′ sites of deoxyribose and its mode of action is dependent on coordinating transition metal ions (e.g. Fe^2+^) where, in the presence of molecular oxygen, the redox active form of metallo-bleomycin is released (11). Since DSBs are less readily repaired *in-vivo*, DNA damage by metallo-bleomycin correlates with its therapeutic effect. Copper complexes also promote ROS-mediated DNA damage with 1,10-phenanthroline (Phen) containing compounds (e.g. [Cu(Phen)_2_]^2+^; **Cu-Phen**) along with mixed chelate derivatives [Cu(Phen)(A-A′)]^+^ (A-A′ = aminoacetate; acetylacetonate; or dicarboxylates) showing clinical potential (12–19). Mononuclear **Cu-Phen** complexes are, however, considered promiscuous compared with metallobleomycins since their oxidative mechanism produces single strand breaks (SSBs) by attacking C-1′ deoxyribose sites in the minor groove (7,20–25). Nevertheless, AMNs can be directed to nucleic acid targets as elegantly demonstrated by Cowan *et al.* when an amino terminal copper/nickel (ATCUN) binding peptide, conjugated to an acridine-based ligand, selectively degraded G-quadruplex telomeric DNA in the presence of copper ions (26).

**Figure 1.**
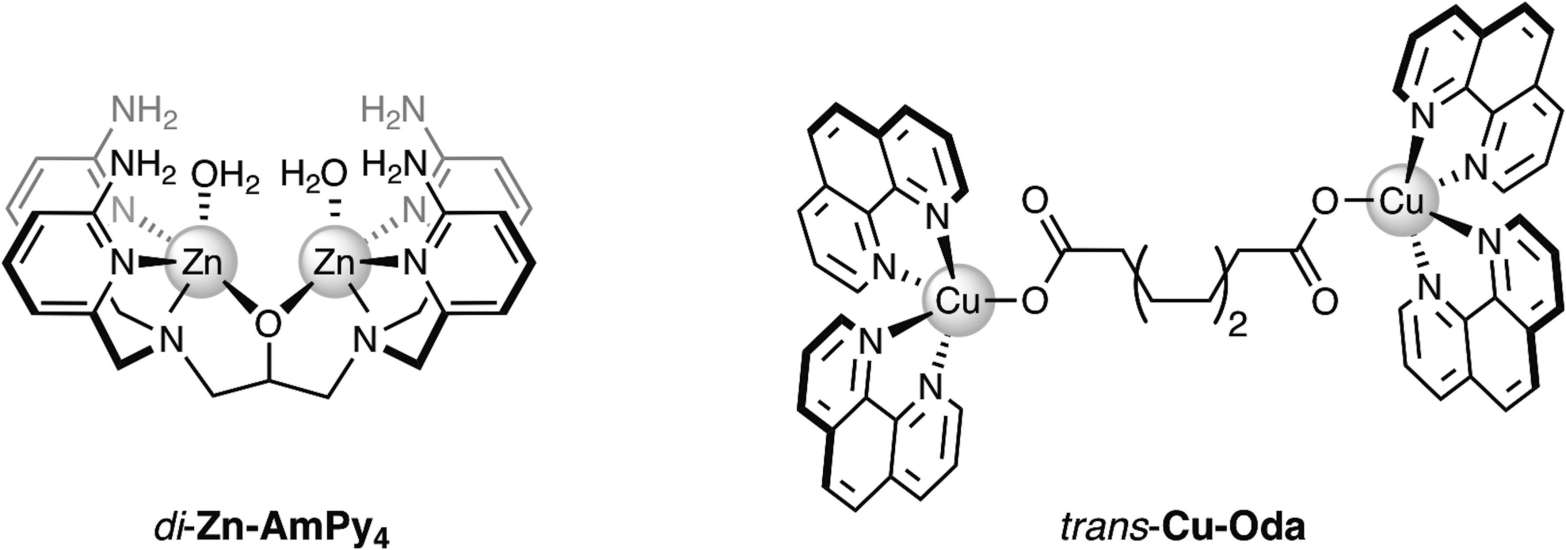
Molecular structures of the bi-metallic Zn^2+^*tetra*-2-aminopyridine complex *di*-Zn-AmPy_4_ and *di*-Cu^2+^ DNA oxidant [Cu_2_(*μ*-octanedioate)(Phen)_4_]^2+^ (*trans*-Cu-Oda).

To develop new artificial metallo-nucleases (AMNs), researchers have focused on introducing two (or more) metal centres into complex scaffolds with particular interest centered on discovering nuclease mimetics that work in the absence of exogenous reductant—so called ‘self-activating’ systems. Several poly-nuclear AMN classes have also shown enhanced DNA cleavage sensitivity together with high sequence selectivity. For example, dinuclear lanthanide Ce^4+^-EDTA and Ce^4+^-EDTP oligonucleotide conjugates (EDTA = ethylenediaminetetraacetic acid; EDTP = ethylenediaminetetrakis-(methylene-phosphonic acid)) were shown to selectively damage G-quadruplex and single stranded DNA targets (27). A *bi*-metallic Zn^2+^ poly-pyridyl complex **Zn_2_-AmPy**_4_ (AmPy = 2-aminopyridine, Figure 1) was subsequently reported to tightly bind and catalytically cleave phosphodiester groups with remarkable efficiency (28). In parallel, Karlin, Rokita and co-workers investigated *di*- and *tri*-Cu^2+^ AMNs coordinated with poly-pyridyl ligands including a *tri*-nuclear copper AMN [Cu_3_(L)(H_2_O)_3_(NO_3_)_2_]NO_3_.2.5H_2_O (L = 2,2′,2″-tris(dipicolylamino)triethyl-amine) with sequence bias at single and double stranded DNA junctions (29,30). A *di*-Cu^2+^ derivative [Cu_2_(D^1^)(H_2_O)_2_]^4+^ (D^1^ = dinucleating ligand with two *tris*-(2-pyridylmethyl)-amine units covalently linked in their 5-pyridyl positions by a -CH_2_CH_2_- bridge) was then reported, and although the same hairpin junction was not targeted, 3′ overhangs were cleaved with higher nucleolytic effects when compared to **Cu-Phen** (31). Later, this group showed that ligand design can switch DNA oxidation sensitivity from deoxyribose to guanine (32) and by modulating inter-metal distances, *di*-Cu^2+^ pyridyl complexes were afforded excellent cleavage sensitivity (33). In addition to these discoveries, it was recently shown that *di*-Cu^2+^ Phen complexes containing bridging dicarboxylate ligands (e.g. [Cu_2_(*μ*-octanedioate)(Phen)_4_]^2+^, *trans*-**Cu-Oda**, Figure 1) can discriminate TA/TA from AT/AT oligonucleotide steps and promote cytotoxicity through a combination of singlet oxygen and superoxide ROS production (34). The nucleolytic profile of these *di*-Cu^2+^ AMNs are also enhanced relative to **Cu-Phen** with both complexes cleaving supercoiled DNA without added reductant.

In this contribution we report the rational design of a new *di*-Cu^2+^ AMN, [Cu_2_(*tetra*-(2-pyridyl)-NMe-naphthalene)Cl_4_] (**Cu_2_TPNap**). Our strategy for designing this synthetic nuclease stems from successful applications of poly-pyridyl ligand scaffolds in Cu^2+^ and Zn^2+^ AMNs. But instead of bridging *bi*-metallic centres with a short alkyl-amino-based linker—commonly employed in polynuclear cleavage agents—we opted to use a rigid aromatic naphthalene linker to facilitate *di*-Cu^2+^ phosphate interactions across the major groove of double stranded DNA. Additionally, we wanted to probe if secondary interactions such as intercalation by the bridging naphthalene moiety were possible and might augment DNA recognition. Herein we report **Cu_2_TPNap** has excellent DNA binding affinity in the order of ∼10^7^ M(bp)^−1^, has exclusive major groove targeting properties, and cleaves DNA through a ‘self-activating’ superoxide-mediated process.

## MATERIALS AND METHODS

CuCl_2_, 2,7-bis(bromomethyl)naphthalene, sodium sulphate and potassium carbonate were purchased from TCI Europe and used without further purification. Bis(2-pyridylmethyl)amine was synthesized as previously reported (35). Deuterated chloroform was obtained from Apollo Scientific. ^1^H NMR spectra were recorded on Jeol ECX-400 and Varian 500 AR spectrometers at room temperature and all chemical shifts are relative to the residual solvent peak. ESI Mass Spectra were recorded on a Waters LCT Premier XE Spectrometer in positive mode. Microanalysis (C, H and N) was carried out using a Perkin Elmer 2400 series II analyzer. UV–Vis spectra were recorded on a Jasco UV–Vis spectrophotometer in phosphate buffer (pH 6.8). Emission experiments were conducted on a Shimadzu RF-5301 spectrofluorometer at room temperature. Thermal melting analysis was conducted on an Agilent Cary 100 dual beam spectrophotometer equipped with a 6 × 6 Peltier multicell system with temperature controller. Circular dichroism spectra were recorded on an Applied PhotoPhysics Chirascan Plus. Fluorescence spectra were recorded on a Perkin Elmer LC55. Calf thymus DNA was purchased from Invitrogen (15633019) while DNA from salmon testes (D1626) and synthetic alternating co-polymers (poly[d(A-T)_2_] (P0883) and poly[d(G-C)_2_] (P9389)) were obtained from Sigma Aldrich Ireland.

### Preparation of ligand and metal complex

#### 2,7-Di(aminomethyl)-N,N,N’,N’-tetra-(2-pyridylmethyl)naphthalene (TPNap)

2,7-bis(bromomethyl)naphthalene (0.300 g, 0.955 mmol) was dissolved in acetonitrile and added to an acetonitrile solution containing *bis*-(2-pyridylmethyl)-amine (0.380 g, 1.9 mmol) and potassium carbonate (0.395 g, 2.86 mmol). The suspension was stirred at room temperature for 3 days and filtered. The solution was then evaporated to dryness, the residue was dissolved in ethyl acetate and washed with water and brine. The organic layer was dried over anhydrous sodium sulphate. Evaporation under vacuum gave a white solid that was purified by column chromatography (silica gel, chloroform/methanol, 9:1). Yield: 0.361 g (68.7%). ^1^H-NMR (CDCl_3_, 400 MHz). 8.51–8.53 (m, 4H), 7.76 (br, s, 2H), 7.52–7.79 (m, 12H), 7.11–7.16 (m, 4H), 3.86 (s, 4H), 3.84 (s, 8H). ^13^C-NMR (CDCl_3_, 400 MHz): 159.87, 149.1, 136.85, 136.66, 136.52, 129.21, 127.8, 127.54, 126.94, 122.92, 122.03, 60.00, 58.73.

#### [Cu_2_(tetra-(2-pyridyl)-NMe-naphthalene)Cl_4_] (Cu_2_TPNap)

A 10 ml methanolic solution of CuCl_2_ (0.067 g, 0.50 mmol) was added to a methanolic solution of **TPNap** (0.138 g, 0.25 mmol) and stirred for 4 h at room temperature. The complex **Cu_2_TPNap** was precipitated by the addition of diethyl ether. Yield: 0.140 g (63%). Elem. Anal. Calcd. (%) for C_36_H_34_N_6_Cl_4_Cu_2_⋅2H_2_O: C, 50.53; H, 4.48; N, 9.82; found: C, 50.29; H, 4.23; N, 9.75. ESI MS: *m/z* calcd. for [**Cu_2_TPNap**-Cl]^+^ 783.04, found 783.05 (100%).

### DNA Binding experiments

#### Competitive ethidium bromide displacement assay

The DNA binding affinity of the title complex was studied in triplicate on ctDNA and synthetic alternating copolymers (poly[d(A-T)_2_] and poly[d(G-C)_2_]) and conducted according to the literature procedure previously reported by Molphy *et al.* (15).

#### Viscosity studies

Experiments were conducted using DV-II-Programmable Digital Viscometer equipped with Enhanced Brookfield UL Adapter at room temperature (∼20°C) by gradually increasing the [compound/DNA] ratios from 0.02 to 0.20 as reported previously (36).

#### Thermal melting studies

Briefly, the concentrations of varying polymers of DNA were determined by measuring absorption intensity at 260 nm using suitable molar extinction coefficients and salt buffered conditions (15,37). Prior to commencing the thermal melting study, **Cu_2_TPNap** (*r* = 0.1) was incubated with DNA for 10 min at 25°C to allow for drug-DNA binding to occur. Melting curves were recorded by monitoring changes in absorbance at 260 nm as a function of temperature from 25 to 95°C. The study was conducted in triplicate and *T*_M_ values were determined from the midpoint of the melting curve or in the case of poly[d(G-C)_2_], by the maxima of the first derivative.

#### Circular dichroism

Complex-DNA interactions were analysed in 10 mM phosphate solution (pH 7.0) in the presence of 25 mM NaCl. Solutions of salmon testes DNA (stDNA, Sigma Aldrich, D1626, ϵ_260_ = 12 824 M(bp)^−1^ cm^−1^), Poly[(d(A-T)_2_] (Sigma Aldrich, PO883, ϵ_260_ = 13 100 M(bp)^−1^ cm^−1^) and Poly[(d(G-C)_2_] (Sigma Aldrich, P9389, ϵ_260_ = 16 800 M(bp)^−1^ cm^−1^) were initially heat treated and allowed to renature prior to quantification using an Agilent Cary 100 dual beam spectrophotometer equipped with a 6 × 6 peltier multicell system with temperature controller to give a working solution with final DNA concentration of ∼100 μM (bp equivalents). The investigation was conducted in the range of 200–400 nm and measurements were recorded at a rate of 1 nm per second. DNA solutions were incubated for 30 min periods in darkness at 37°C with **Cu_2_TPNap** at varing *r* ([drug] / [DNA]) values where *r* = 0.1 and 0.2 (*r* being the ratio of complex per μM DNA).

#### bis-(2,4-Dinitrophenyl)phosphate (BDNPP) cleavage assay

Reactions were carried out according to the literature procedure reported by Coleman *et al.* with minor changes (38). The hydrolysis rate of BDNPP was measured by monitoring the increase in visible absorbance at 400 nm due to the release of the 2,4-dinitrophenolate anions. Metal complex solutions were buffered with 50 mM PIPBS (pH 3.5–5), MES (pH 5–6.7), HEPES (pH 6.8–8.5) and CHES (pH 8.5–11.0). The ionic strength was maintained at 0.1 M with KNO_3_. Rate constants were obtained by the initial rate method (<5% conversion). The concentration of 2,4-dinitrophenolate was calculated from the extinction coefficient (12 100 M^−1^ cm^−1^). The concentrations were corrected for the degree of ionization of 2,4-dinitrophenol at the respective pH value using p*K*_a_ (2,4-dinitrophenol) = 4.0 (39). In a typical experiment 15 μl of a freshly prepared BDNPP stock solution (5 mM in DMSO) was added to a solution of the metal complex (1.5 ml, 0.25–5 mM) at 40 °C. Cleavage rates have been corrected for the spontaneous hydrolysis of the substrate (40).

### DNA damage studies

#### DNA cleavage studies

Reactions were carried out according to the literature procedure reported by Kellett *et al.* with minor changes (41). DNA-complex incubation was carried out at 37°C for 1 h in the absence of exogenous reductant. Samples were loaded onto an agarose gel (1.2%) and electrophoresis was completed at 70 V for 1 h in 1× TAE buffer. Gels were visualized and photographed on a UVP laboratory products Epi Chemis II Darkroom transilluminator.

#### Hydrolytic DNA damage investigation using T4 ligase

Reactions were adapted from the literature procedure reported by Prisecaru *et al.* (41). 800 ng pUC19 was initially treated with 60 μM of **Cu_2_TPNap** in the presence of 25 mM NaCl for 5 h at 37°C. Control linear (LC) DNA was obtained through the treatment of 400 ng of pUC19 with type II restriction endonuclease EcoRI (NEB, R0101S) for 1 h at 37°C followed by heat denaturation for 20 min at 65°C. Gel electrophoresis was carried out and linear DNA (LC) bands were excised, purified and eluted in 30 μl nuclease free H_2_O using QIAquick PCR purification Kit (Qiagen, 28104). Purified DNA was incubated with 2 μl T4 DNA Ligase (NEB, M0202S) and 3 μl of T4 DNA Ligase Reaction Buffer (10×) in an ice bath overnight and slowly brought to room temperature.

#### DNA alkylation inhibition investigation—Melphalan assay

This assay was adapted from the literature procedures previously reported by Wang *et al.* and Farrell *et al.* (42,43). The pBR322 vector (4361 bp) was selected and a set of primers (Forward: 5′-GCTGCAAAACGTCTGCGACC-3′ and reverse: 5′-CGCATCAGGCGCTCTTCCGC-3′) were designed to generate an amplicon containing 798 bp (54% GC content). The fragment was generated by carrying out PCR (35 cycles) with 1 ng of pUC19 plasmid with 2× MyTaq Red Mix (Bioline) and identified using gel electrophoresis with a 1 Kb DNA ladder (Fermentas) as control. 400 ng of the fragment was then incubated with 0.10, 0.25 and 0.50 mM **Cu_2_TPNap** for 30 min at 37°C. The complex-DNA solution was then exposed to 5 μM of melphalan for 30 min at 37°C followed by DNA heat denaturation for 15 min at 90°C. 2 μl of a 1 M piperidine solution was then added and incubated for 1 h at 90°C. Control experiments were conducted with 400 ng of DNA exposed to 0.05, 0.10, 0.25 and 0.50 mM of netropsin under identical conditions.

#### Competitive binding of mithramycin A with poly[d(G-C)_2_] in the presence of Cu_2_TPNap

This assay was adapted from a procedure previously reported by Farrell *et al.* (37). Stocks of mithramycin A (Sigma Aldrich, M6891, ϵ_400_ = 10 000 M^−1^ cm^−1^) and poly[d(G-C)_2_] (ϵ_254_ = 8400 M^−1^ cm^−1^) were prepared in DMF and NF H_2_O respectively and concentrations were determined spectrophotometrically. A 100 μM solution of poly[d(G-C)_2_] in 10 mM PO_4_^3−^, 50 mM NaCl and 10 mM MgCl_2_ (pH 7.4) was incubated for 15 minutes at RT in the dark with Cu_2_TPNap at a ratio of 0.10 drug/nucleotide. Varying amounts of mithramycin A (*r* = 0–0.25) were then added to complex treated and complex untreated poly[d(G-C)_2_] solutions and allowed to incubate in the dark for 5 minutes prior to analysis. Emission spectra were recorded in the range 525 – 625 nm with an excitation wavelength of 470 nm to avoid photodegradation (excitation slit: 2.5 nm/emission slit: 5 nm). Triplicate values were recorded and all data was analysed at 550 nm.

#### DNA cleavage in the presence of ROS scavengers

Reactions were adapted from a literature procedure recently reported by this group (44). To a final volume of 20 μl, 80 mM HEPES (pH 7.2), 25 mM NaCl and 400 ng pUC19 DNA were treated with 5, 10, 20 and 40 μM of **Cu_2_TPNap** in the presence of ROS scavengers; dimethylsulfoxide (DMSO, 10%), 4,5-dihydroxy-1,3-benzenedisulfonic acid (Tiron, 10 mM), pyruvate (10 mM) and sodium azide (NaN_3_, 10 mM). Reactions were incubated for 1 h at 37°C and subjected to electrophoresis.

#### HT Quantitation of 8-oxo-dG

Quantitation of 8-oxo-dG lesions present in 3000 ng pUC19 plasmid DNA pre-incubated for 30 min with **Cu_2_TPNap** (40 and 60 μM) and 1 mM of Na-l-ascorbate at 37°C was achieved utilizing a high throughput 8-oxo-dG ELISA kit (Trevigen) and performed as per manufacturer's guidelines. Experimental procedure was carried out in triplicate as previously reported by Molphy *et al.* (44).

#### DNA cleavage of single stranded M13mp18 DNA

Briefly, cleavage reactions were conducted on 400 ng single stranded M13mp18 plasmid DNA (7249 bp, NEB, N4040S) in the presence of 25 mM NaCl and exposed to increasing concentrations of **Cu_2_TPNap** (5, 10, 20, 40 μM) for 30 min at 37°C and subjected to gel electrophoresis on a 0.8% agarose gel at 60 V for 70 min. EcoRI was chosen as a control due to its restriction site at 6230 bp on the circular vector.

#### Pre-associative molecular docking studies

The dockings of the di-copper complex with chosen DNA fragments were modelled using AutoDock (Version 4.2.5) (45). The molecular structure of **Cu_2_TPNap** was obtained from single-crystal X-ray diffraction data (Supplementary Figure S-1) and hydrogen atoms were located at their calculated positions. The structure of the DNA fragment d(GGGGCCCC)_2_ (PDB: 2ANA) (46) was obtained from the Brookhaven Database. These structures did not include hydrogen atoms, and these were added at their calculated positions using GaussView 3.0. The Gaussian 09 newzmat utility was then used to generate the .pdb input files as required by AutoDock. Analysis of the tortional freedoms in the ligands confirmed that [Cu_2_(*tetra*-(2-pyridyl)-naphthalene)Cl_4_] contains four tortional degrees of freedom. The molecular structures of [Cu_2_(*tetra*-(2-pyridyl)-naphthalene)Cl_3_]^+^ and [Cu_2_(*tetra*-(2-pyridyl)-naphthalene)Cl_2_]^2+^ were calculated using molecular mechanics methods (UFF) as implemented in Gaussian 09 (Revision E.01) (47). In each docking the search space encompassed the entire receptor which was treated as a rigid structure. The docking calculations used the Lamarcian Genetic Algorithm (48). The docking studies provided an initial ligand acceptor structure for the 2ANA-[Cu_2_(tetra-(2-pyridyl)-naphthalene)Cl_4_] interaction, and this was then used as a starting geometry for further optimization using the ONIOM approach as implemented in the Gaussian 16 program suite (Revision B.01) (49–54). In these calculations the ANA fragment was treated at a low level of theory i.e. Molecular Mechanics MM (47) while the copper complex was modelled using the B3LYP hybrid DFT functional (55,56) and the LanL2DZ basis set (57).

## RESULTS AND DISCUSSION

### Preparation of the *di*-copper(II) complex

**Figure 2.**
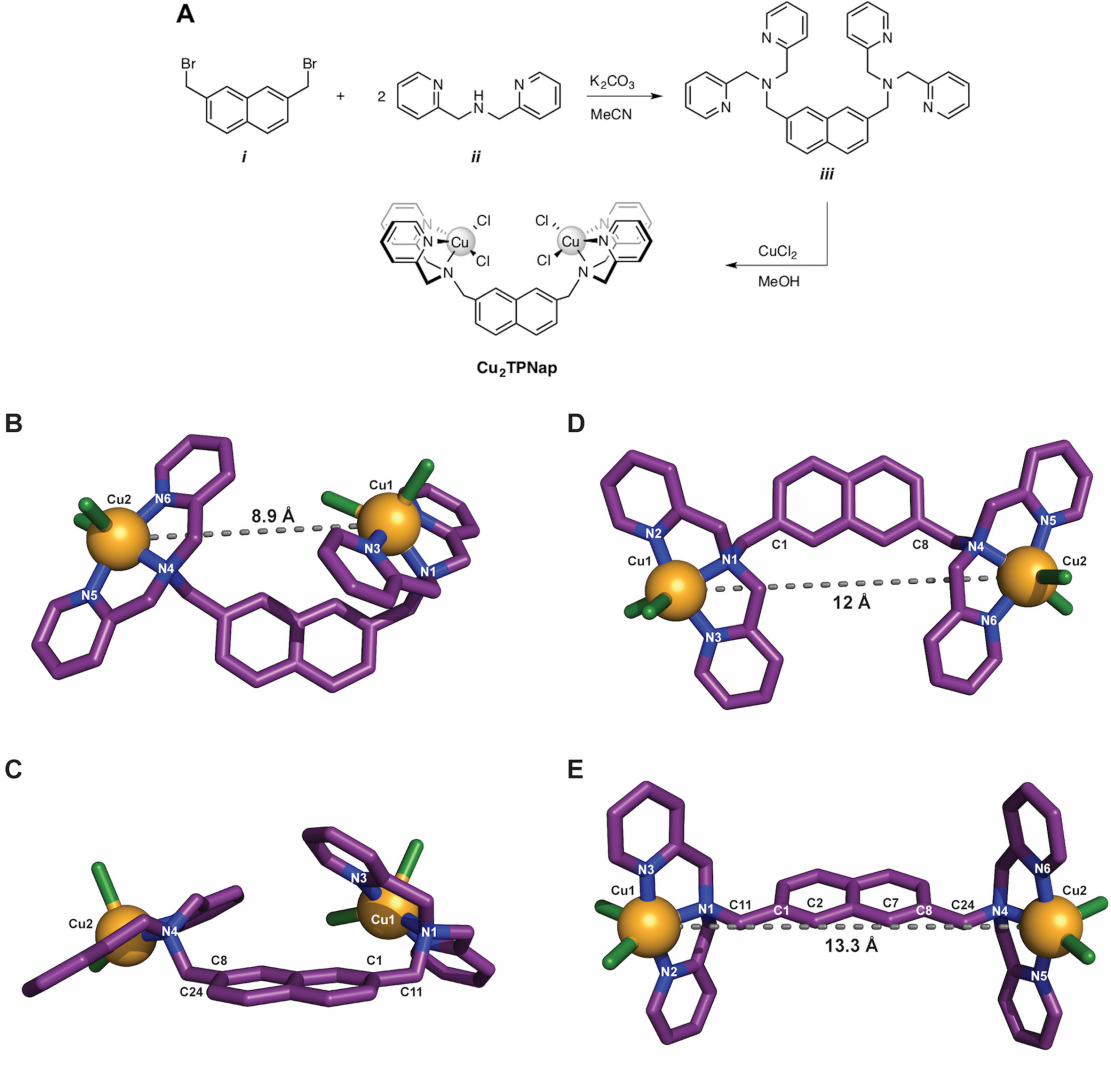
(**A**) Synthetic route toward generating the Cu_2_TPNap complex; (**B**) X-ray structure showing a perspective view of Cu_2_TPNap where copper ions are bridged by the naphthalene-methylamine group and the intermetal Cu•••Cu distance is 8.9 Å; (**C**) alternative perspective of Cu_2_TPNap; (**D**) rotation around methylamine bonds to their maximum dihedral angle of 180° resulting in a 12 Å intermetal distance and (**E**) further 180° rotation about methyl-naphthalene bonds yielding a maximum distance of 13.3 Å between Cu•••Cu centres. Figures B-E were generated using the PyMOL Molecular Graphics System, Version 2.0 Schrödinger, LLC. Colour scheme: copper, light orange; carbon, violet purple; nitrogen, deep blue; chloride, split pea.

The *tetra*-(2-pyridyl)-NMe-naphthalene (TPNap) ligand was generated by the addition of two equivalents of *bis*-(2-pyridylmethyl)-amine to a solution of 2,7-*bis*-(bromomethyl)-naphthalene in acetonitrile containing excess potassium carbonate. Subsequent treatment by CuCl_2_ in methanol generated **Cu_2_TPNap** (Figure 2A) from which crystals suitable for X-ray analysis were grown upon slow evaporation of acetonitrile at room temperature (Figure 2B). Each copper atom in **Cu_2_TPNap** is five-coordinate with an N_3_Cl_2_ donor set. The coordination geometry is best described as distorted square pyramidal with a chloride anion occupying the apical position (see Supplementary S-1 for full crystallographic data). The N–Cu–N and Cl–Cu–Cl bite angles are 80.80(9)° and 105.71(3)° respectively, with the naphthalene scaffold holding the two metal centres 8.9 Å apart (Figure 2B and C). There are four torsional degrees of freedom in the complex centered about the methyl groups connecting the metal-coordinated tertiary amine and naphthalene rings (C11 and C24 in Figure 2C). Rotation about the methylamine bonds to their maximum dihedral angle (180°) in Cu1/N1/C11/C1 and Cu2/N4/C24/C8 sets is shown in Figure 2D and separates the inter-metal distance by ∼12 Å. Further rotation about the methyl-naphthalene bonds, where N1/C11/C1/C2 and N4/C24/C8/C7 dihedrals are opened to 180°, yielded a maximal distance between the copper centres of ∼13.3 Å (Figure 2E).

### DNA binding studies involving fluorescence, absorbance, and thermal melting

**Figure 3.**
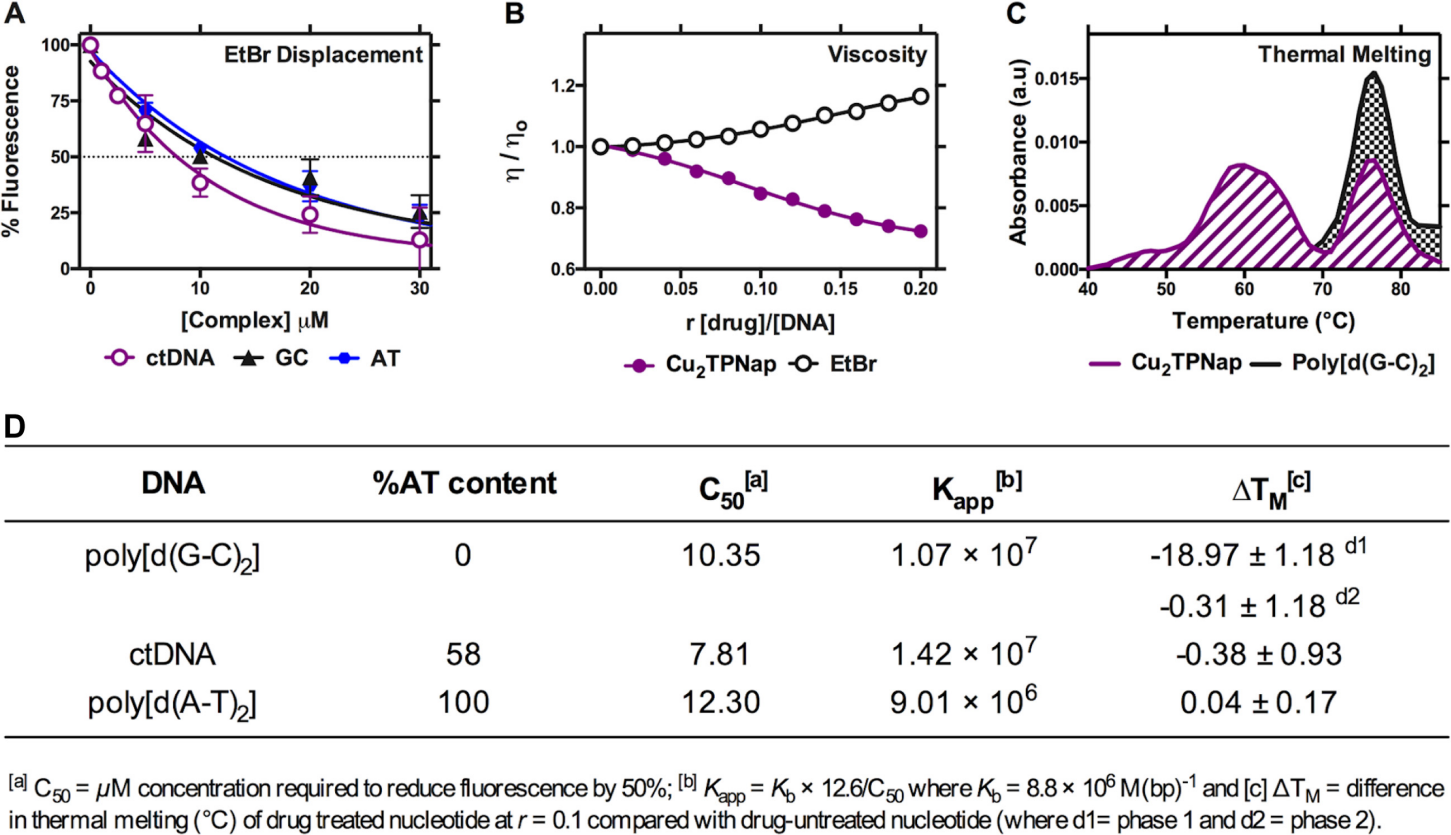
(**A**) Binding of Cu_2_TPNap complex to ethidium-saturated solutions of dsDNA (ctDNA, poly[d(A-T)_2_] and poly[d(G-C)_2_]); (**B**) viscosity profile of complex treated and EtBr treated salmon testes dsDNA; (**C**) thermal melting profile of untreated poly[d(G-C)_2_] nucleotide and complex treated nucleotide at *r* = 0.1; and (**D**) apparent DNA binding constants (*K*_app_) and influence on thermal denaturation of Cu_2_TPNap.

The DNA binding constant of **Cu_2_TPNap** was determined using high-throughput saturation binding analysis with the fluorogenic intercalator ethidium bromide (EtBr) and a selection of duplex DNA polymers (Figure 3A). The complex is a high-affinity DNA binder with *K*_app_ (apparent binding constant) values of *ca*. 1 × 10^7^ M(bp)^−1^ with calf thymus DNA (ctDNA) and poly[d(G-C)_2_], while a slightly lower equilibrium constant was observed with poly[d(A-T)_2_]. To our knowledge these *K*_app_ binding values are amongst the highest reported in the literature and are broadly in line with Cu^2+^ phenanthrene complexes containing designer phenazine-type intercalators (15). **Cu_2_TPNap** was found to quench saturated EtBr bound DNA polymers without adenine-thymine (A-T) specificity and displaced both limited bound EtBr (intercalator) and Hoechst 33258 (minor groove binder) from ctDNA with similar affinity (Supplementary S-2). In order to help classify the binding interaction with DNA, viscosity experiments with double stranded salmon testes DNA (stDNA) were employed (Figure 3B). Here, the complex was identified to condense DNA with a significant reduction in the hydrodynamic profile that contrasted with the classical intercalator EtBr where DNA underwent unwinding and lengthening. In an effort to delineate **Cu_2_TPNap** from the classical intercalator EtBr further, topoisomerase I unwinding experiments with pUC19 DNA showed no evidence of intercalation over a tested concentration range where EtBr is fully active (data not shown). Following this observation, UV melting (*T*_M_) experiments were conducted (Figure 3C) using identical DNA polymers to those employed in the EtBr displacement study (Figure 3D). Interestingly, no stabilization of ctDNA or poly[d(A-T)_2_] was afforded by **Cu_2_TPNap** but the complex was found to destabilize poly[d(G-C)_2_]. Here, a biphasic thermal melting profile was observed with the first derivative (d^1^) melting at a highly negative value of -18.5 ± 1.2°C, and the second (d^2^) at –1.5 ± 1.2°C (Figure 3C). These results depart substantially from classical non-covalent minor groove and intercalating agents netropsin and actinomycin D (15). Additionally, we conducted a parallel study with the major groove binder methyl green where stabilization of +5.2°C was observed with poly[d(G-C)_2_] (Supplementary S-2).

### Circular dichroism analysis

**Figure 4.**
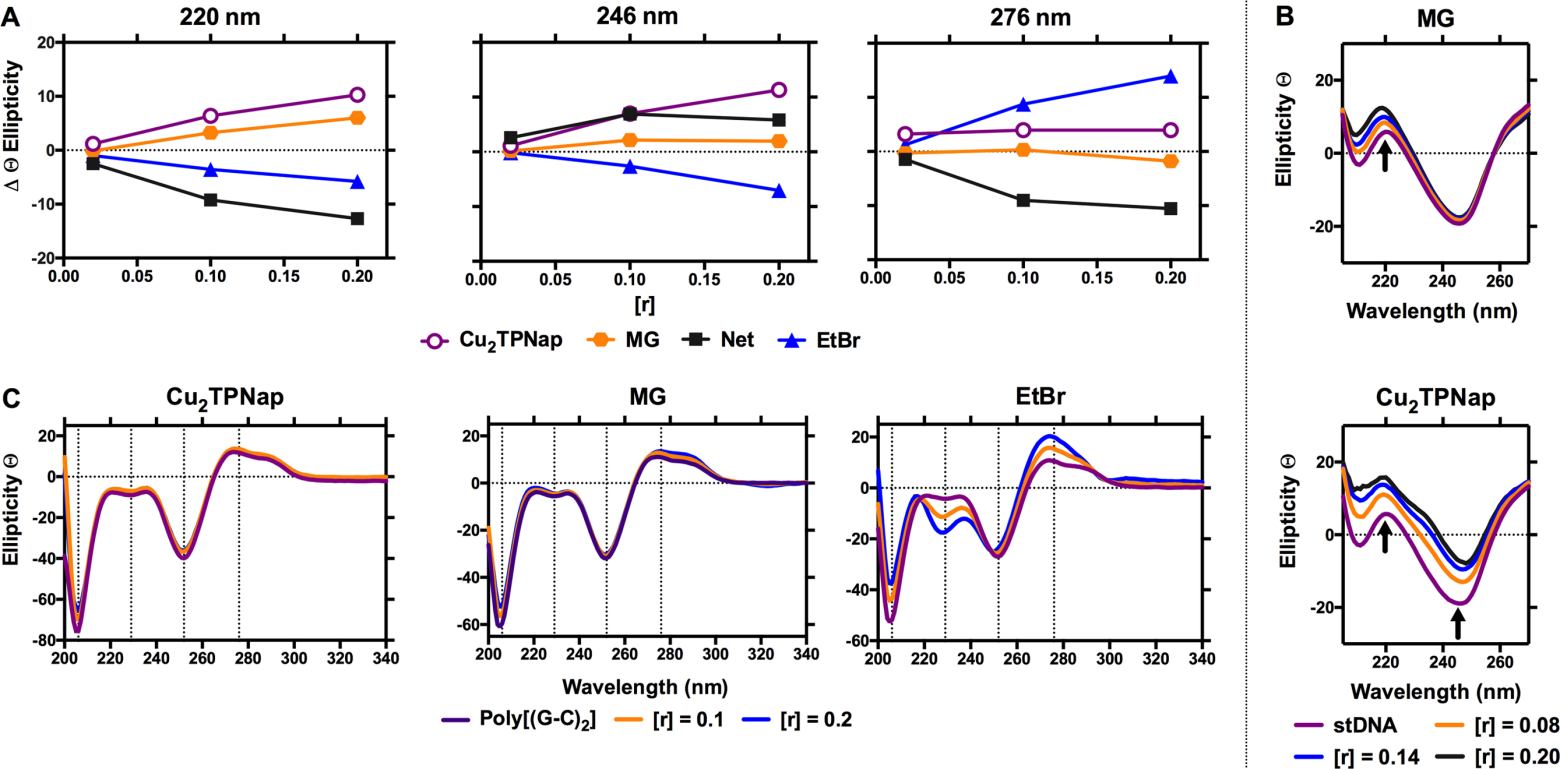
(**A**) Change in ellipticity of Cu_2_TPNap and classical major groove (MG), minor groove (net) and intercalating agent (EtBr) with respect to classical B-form stDNA at *r* = 0.1 and 0.2 loading ratios at 220, 246, and 276 nm; (**B**) increasing ratios of MG and Cu_2_TPNap on stDNA; and (**C**) interactions of Cu_2_TPNap, MG and EtBr on alternating copolymer poly[d(G-C)_2_].

To probe drug-induced conformational changes within duplex DNA, circular dichroism (CD) spectroscopy studies were undertaken. DNA polymers were monitored at 220 nm (hydrogen bonding), 246 nm (handedness/helicity) and 276 nm (base pair stacking) in the presence of **Cu_2_TPNap** and compared to methyl green (MG; major groove binder), netropsin (Net; minor groove binding) and EtBr (intercalator). **Cu_2_TPNap** induced broadly similar spectral changes to stDNA as MG (Figure 4A) where an increase in hydrogen bond distance linking individual base pairs (220 nm) and negligible base pair stacking interactions (276 nm) were observed (Figure 4B). Differences between **Cu_2_TPNap** and MG did arise at 246 nm however, where an increase in ellipticity was observed. This result points toward the complex perturbing the secondary DNA structure with an associated loss of helicity. Interestingly, the complex induced almost identical CD changes in poly[d(G-C)_2_] as MG with increased ellipticity at 210 nm and no appreciable changes at both 246 and 276 nm (Figure 4C). This interaction was quite different to EtBr, which altered the spectra in line with its propensity to intercalate (Figure 4C). Furthermore, the binding interaction of **Cu_2_TPNap** was distinct to Net where stDNA and poly[d(A-T)_2_] polymers showed base stacking reductions (276 nm) due to Net-induced minor groove contraction. Overall, structural changes observed here support a non-intercalative binding profile by **Cu_2_TPNap** that is, in part, similar to the non-covalent binding interaction of MG. The complex-induced changes to helicity—visible at 246 nm in salmon testes DNA (Figure 4B) and poly[d(A-T)_2_] (Supplementary S-2)—are not observed in the presence of MG suggesting **Cu_2_TPNap** binding induces structural distortion to both stDNA and poly[d(A-T)_2_]. But since there is a lack of perturbation to the handedness of poly[d(G-C)_2_], a well-matched binding interaction with **Cu_2_TPNap** seems likely.

### Melphalan protection and mithramycin A competition assays

**Figure 5.**
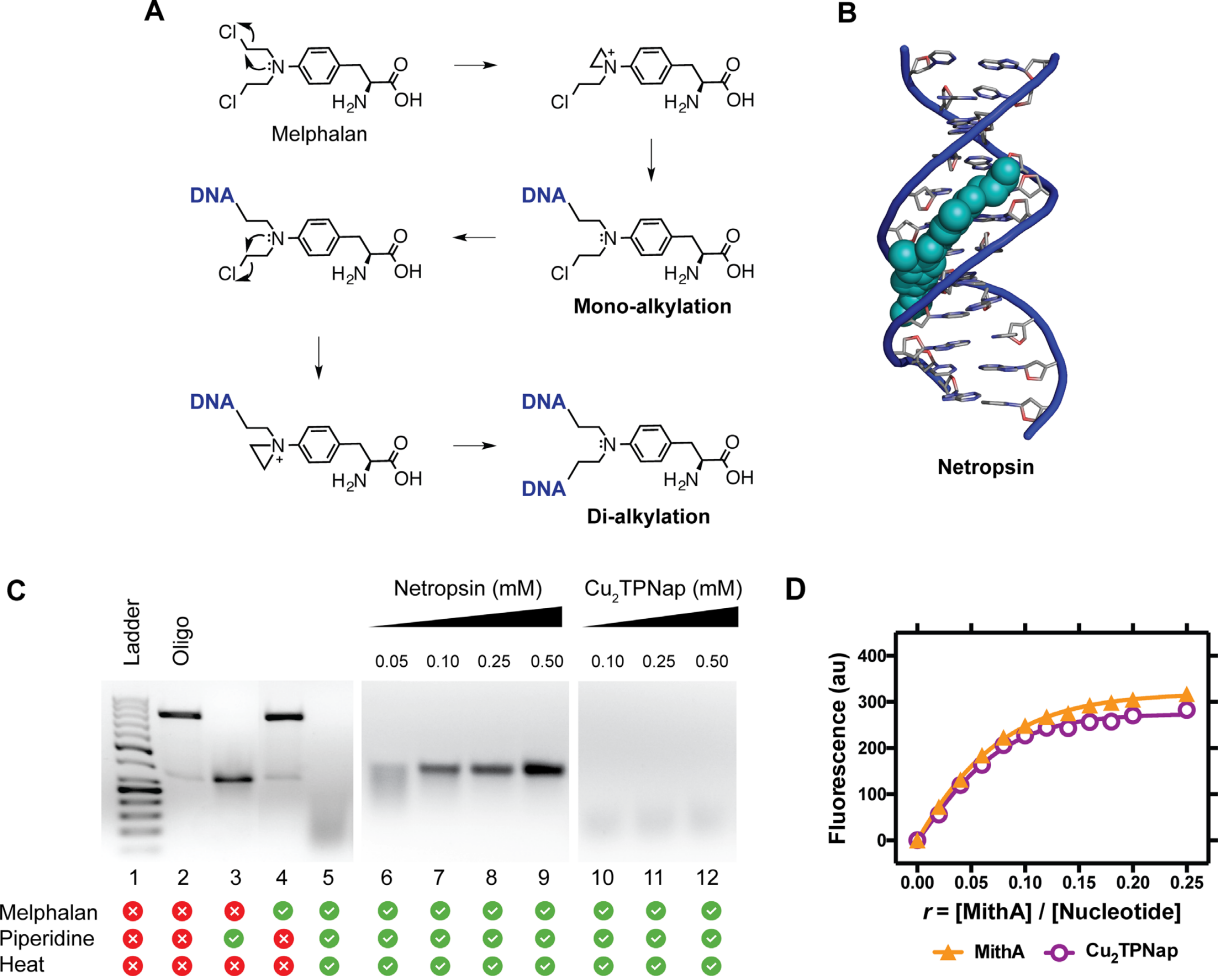
(**A**) Mechanism of DNA alkylation by nitrogen mustard melphalan; (**B**) minor groove binding agent netropsin binding to the minor groove of Dickerson Drew dodecamer (PDB 4C64); (**C**) melphalan protection assay with netropsin (lanes 6–9) and Cu_2_TPNap (lanes 10–12) and (**D**) fluorescence binding of mithramycin A (MithA) to poly[d(G-C)_2_] in the presence and absence of Cu_2_TPNap (*r* = 0.10).

To help establish the major groove as the recognition site of **Cu_2_TPNap**, two indirect assays were selected. In the first case, a melphalan protection assay was employed. The labile chlorine leaving groups in melphalan facilitate nucleophilic attack of nitrogen to form the imminium ion creating a strained ring system (aziridine ion) which readily undergoes alkylation to firstly form a mono-alkylated adduct. This process can be repeated to form a *di*-alkylated adduct which results in DNA crosslinking between two complementary strands (interstrand, Figure 5A) or within a single DNA strand (intrastrand). Melphalan can alkylate to both guanine N7 (major groove) and adenine N3 (minor groove) producing thermolabile sites which are visualized by gel electrophoresis as DNA cleavage upon treatment with piperidine (42,43,58,59). The presence of ligands with a strong minor groove binding capacity (e.g. distamycin) is known to protect the minor groove binding sites from the effects of alkylation. To identify if **Cu_2_TPNap** could offer protection to the minor groove in a similar manner as netropsin (Figure 5B)—used here as a control—an oligonucleotide fragment of 798 bp was generated by the polymerase chain reaction (PCR) containing 54% GC content along with four CpG islands. The amplicon was exposed to melphalan and heat-treated with piperidine to promote thermolabile DNA fragmentation (Figure 5C, lane 5). Pre-exposure of this oligomer to netropsin (0.05–0.5 mM) resulted in concentration-dependent protection of the oligomer (Figure 5C lanes 6–9). **Cu_2_TPNap** was found to offer no protection to the oligonucleotide across the same concentration range, which potentially supports the major groove as the recognition site for this complex (Figure 5C, lanes 10–12). To further establish the major groove as the recognition site, a second assay involving the use of mithramycin A (MithA) was employed. This agent is an aureolic acid-type antibiotic produced by several streptomycete species and is composed of a tricyclic aromatic polyketide core moiety bound to two oligosaccharide chains (60). In the presence of bivalent cations such as Mg^2+^, sequence selective DNA binding by the MithA dimer is attributed to direct hydrogen bonding between the OH-8 group of the antibiotic and the 2 amino group of guanine at GpG (or CpC) sites, while the saccharide chains of MithA wrap the minor groove (61–65). Since MithA binds the minor groove of G-C rich sequences, we employed this molecule as a competitive fluorescence probe with poly[d(G-C)_2_] to gain further insight into the binding mode of **Cu_2_TPNap**. Pretreatment with the dinuclear complex (*r* = 0.10 [drug]/[nucleotide]) produced negligible change in MithA binding that did not depart substantially from the control experiment involving MithA and poly[d(G-C)_2_] alone (Figure 5D). Taken together, these results suggest **Cu_2_TPNap** does not appear to have significant minor groove residency in B-DNA or poly[d(G-C)_2_].

### Preassociative docking studies

**Figure 6.**
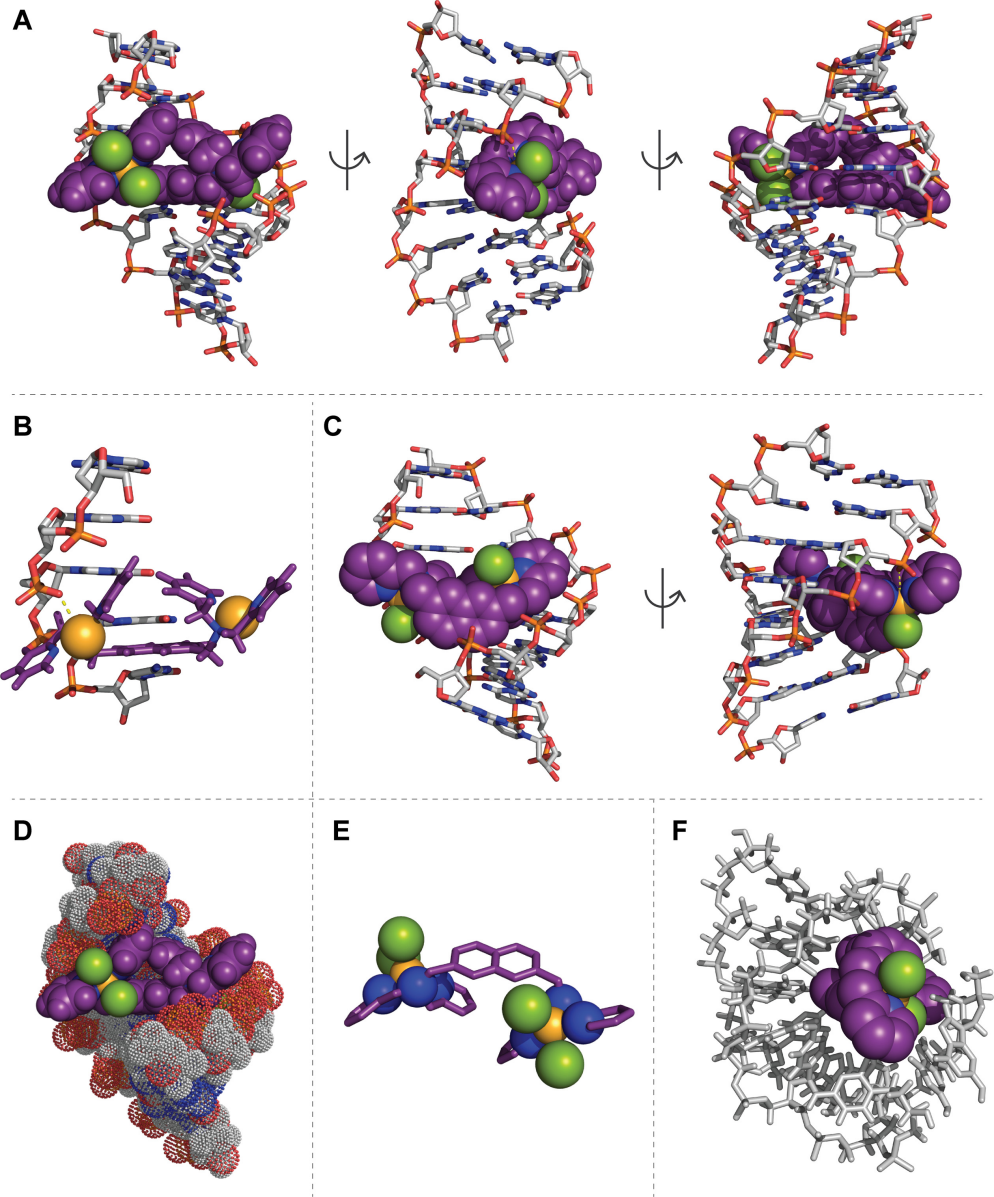
(**A**) Cu_2_TPNap docks with d(GGGGCCCC)_2_ (PDB 2ANA) in the major groove with the naphthalene ring parallel to DNA base pairs; (**B**) coordination of copper(II) in Cu2TPNap with a phosphate oxygen atom at the second GpG step (chlorides removed for clarity); (**C**) Cu_2_TPNap^2+^ dication docking with d(GGGGCCCC)_2_ in the major groove where the naphthalene ring orients orthogonal to DNA bases; (**D**) space filled view of the neutral Cu_2_TPNap docked with 2ANA (nucleic acid backbone shown as dots); (**E**) symmetry of the neutral complex when docked with 2ANA; and (**F**) analysis of the adduct DNA-metal complex using a two-level ONIOM calculation whereby the d(GGGGCCCC)_2_ sequence relaxes about the di-Cu^2+^ complex. Figures generated using the PyMOL Molecular Graphics System, Version 2.0 Schrödinger, LLC. Complex colour scheme: copper, lightorange; oxygen, red; carbon, violetpurple; nitrogen, deepblue; chloride, splitpea; hydrogen; white.

Given the thermal melting and CD results observed with poly[d(G-C)_2_], together with evidence of selective major groove recognition, a pre-associative docking study was undertaken with the 8-mer d(GGGGCCCC)_2_ (PDB: 2ANA), which was initially treated as a rigid receptor. This sequence is a good model for poly[d(G-C)_2_] as it contains a wide shallow minor groove and a deep major groove approximated at 12.6 Å wide (46). The neutral **Cu_2_TPNap** complex was identified to dock into the major groove with one Cu(II) ion coordinating to a phosphate oxygen atom at the second GpG step (Figure 6A, Movie_S1). The Cu•••O(P) contact is 3.2 Å long and the complex adopts a *trans* configuration about the naphthalene ring which orientates parallel to the bases of the GpC step (Figure 6B). In the *di*-cation, where the removal of a bound chloride ion from each metal centre was simulated, the complex is also in a *trans* configuration (Movie_S2) but this time the naphthalene ring is orthogonal to the DNA bases (Figure 6C) with the same Cu(II) ion coordinated to the second GpG step with a longer Cu•••O(P) contact (3.6 Å). A spacefilling model of the docked **Cu_2_TPNap** complex is shown in Figure 6D and provides additional support to earlier CD analysis for the complex being well matched to the major groove of the d(GGGGCCCC)_2_ oligomer. In terms of complex geometry, docking of the neutral **Cu_2_TPNap** complex results in an inter-metal copper(II) distance of 9.8 Å while the *di*-cation inter-metal distance was shorter at 8.9 Å (Figure 6E).

To further describe the binding interaction of **Cu_2_TPNap** with 2ANA and to help probe the stability of the adduct DNA–metal complex, a two-level ONIOM calculation was undertaken. In this calculation, the copper complex was treated at a high level of theory, namely B3LYP/LanL2DZ while the 2ANA sequence was optimized using a low level UFF molecular mechanics approach. The d(GGGGCCCC)_2_ sequence was allowed to relax about the *di*-Cu^2+^ complex and the optimization steps showed the collapse of 2ANA around the complex. These optimization steps are shown in Movie_S3. In the relaxed structure, Watson–Crick pairing is ablated and the double helical structure is no longer evident, with both nucleic acid chains winding around the complex (Figure 6F). Broad support for this interaction can be found from UV melting and viscosity experiments where significant destabilization of poly[d(G-C)_2_] along with condensation of salmon testes dsDNA fibres were identified.

### Artificial chemical nuclease activity

**Figure 7.**
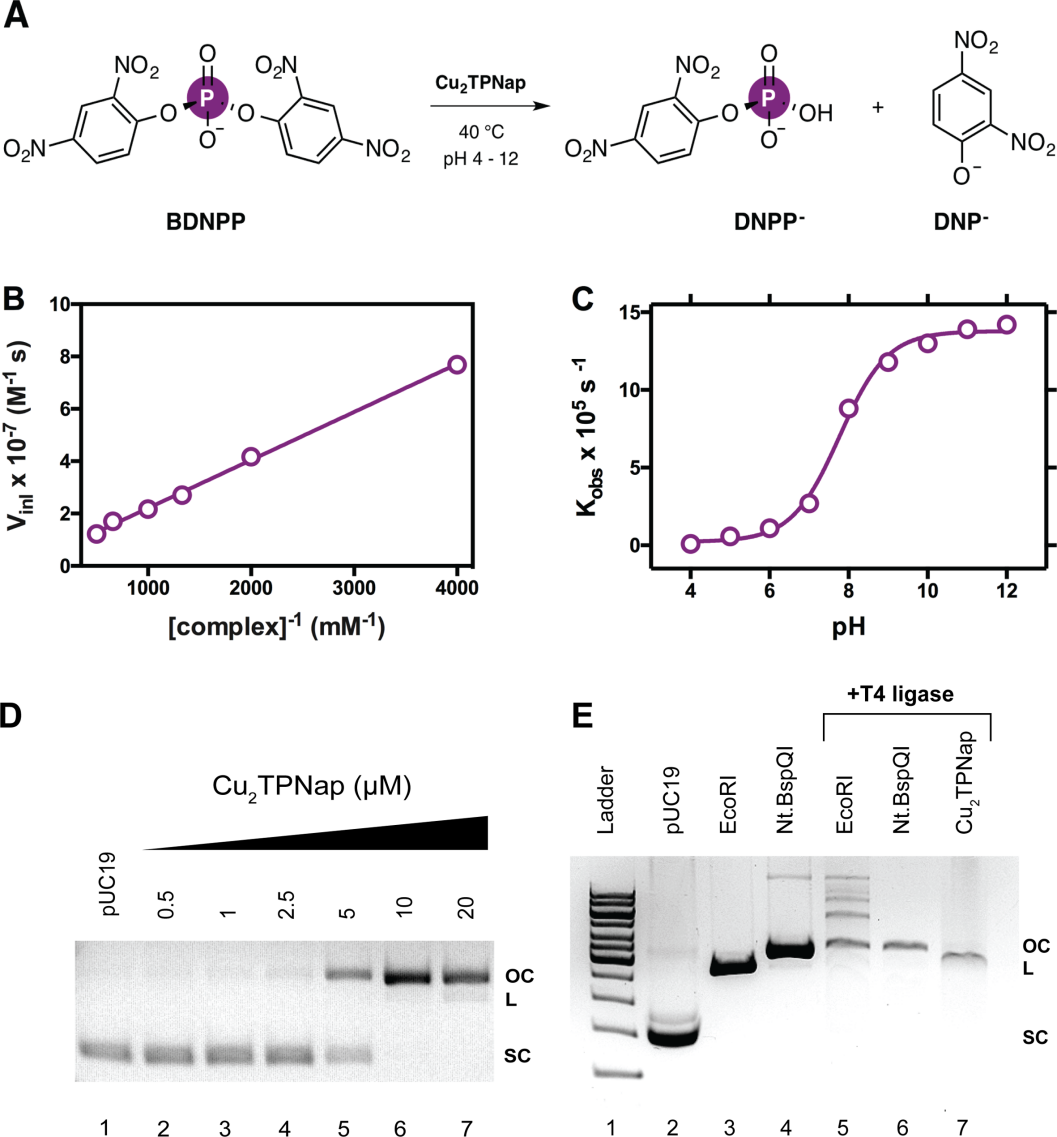
(**A**) BDNPP hydrolytic cleavage mechanism in the presence of Cu_2_TPNap; (**B**) Lineweaver–Burk plot; (**C**) rate-pH profile for the cleavage of BDNPP in the presence of Cu_2_TPNap at 40°C; (**D**) DNA cleavage reactions by Cu_2_TPNap on pUC19 plasmid DNA over 1 h at 37°C in the absence of added reductant; and (**E**) T4 DNA ligase experiments with Cu_2_TPNap and restriction enzymes EcoRI and Nt.BspQI.

To identify **Cu_2_TPNap**-mediated phosphodiesterase activity, hydrolysis of the model substrate *bis*-(2,4-nitrophenyl)-phosphate (BDNPP) was examined. The hydrolysis rate of BDNPP with the complex was measured spectrophotometrically where the release of the chromophore 2,4-dinitrophenolate (Figure 7A) was identified at 400 nm. The linear Lineweaver-Burk plot indicated Michaelis-Menten behaviour with formation of a kinetically active complex-substrate intermediate (Figure 7B). From this plot, the hydrolysis rate of the bound substrate (*k*_cat_ = 5.4 × 10^−3^ s^−1^), the maximum rate (*V*_max_ = 2.7 × 10^−7^ M s^−1^), and the substrate binding constant (1/*K*_m_ = 205 M^−1^) were obtained and indicate hydrolytic activity at the higher end of the range typically observed for dinuclear copper(II) complexes. The pH dependence of the reaction rate of BDNPP cleavage was studied over the range 4.0–12.0. The plot of the pseudo-first order rate constant versus pH revealed a sigmoidal curve with an inflection point at pH 8.2 that corresponds to the kinetic p*K*_a_ value (Figure 7C). To identify artificial nuclease activity, supercoiled (SC) pUC19 DNA was then examined in the presence of the dinuclear complex. Here, single-strand nicking to the open circular form (OC) was identified in a concentration-dependent manner in the absence of exogenous factors (*i.e*. without added peroxide or reductant) with stepwise conversion of single-strand to double-strand breaks (Figure 7D) supporting DSB formation from two independent nicking events (66). Since **Cu_2_TPNap** displayed self-activating cleavage properties and efficiently hydrolysed BDNPP, it was important to explore the nature of DNA excision. Hydrolytic and oxidative DNA damage can be distinguished through the use of T4 DNA ligase—an enzyme that catalyses the religation of hydrolytically cleaved DNA duplexes *via* phosphodiester bond formation (67). Linear plasmid DNA restricted by **Cu_2_TPNap** in the absence of reductant (Supplementary S-2) was excised from agarose, purified, and then treated with T4 DNA ligase. Control experiments were conducted in parallel using the type II restriction endonuclease EcoRI, which has one recognition site on the pUC19 vector (Figure 7E, lane 3), and also with the single stranded nicking enzyme Nt.BspQI (Figure 7E, lane 4). Interestingly, **Cu_2_TPNap** restricted DNA could not undergo religation when treated with T4 ligase potentially indicating a non-hydrolytic DNA cleavage mechanism (Figure 7E, lane 7). This result contrasted with EcoRI transformed pUC19, which formed open circular DNA and larger concatamers (Figure 7E, lane 5) indicative of DNA religation by hydrolytic excision with Nt.BspQI nicked DNA subsequently treated with T4 ligase serving as an effective secondary control where DNA was maintained in its open circular form (Figure 7E, lane 6).

### Oxidative DNA damage mechanism

**Figure 8.**
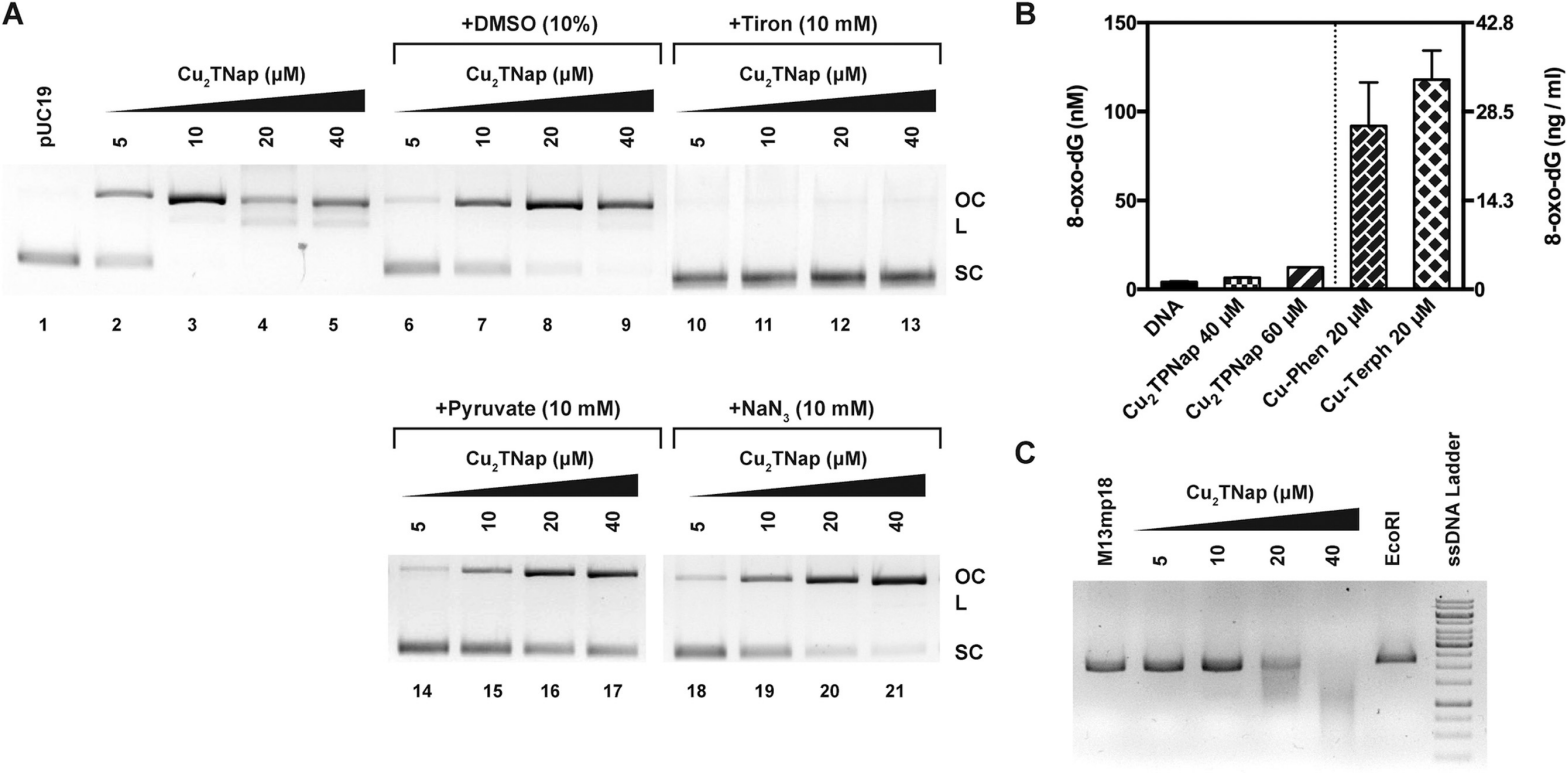
(**A**) Cu_2_TPNap DNA cleavage experiments in the absence (lane 2–5) and presence of free radical antioxidants including DMSO (^•^OH, lane 6–9), tiron (O_2_^•−^, lane 10–13), pyruvate (H_2_O_2_, lane 14–17) and sodium azide (^1^O_2_, lane 18–21); (**B**) quantification of 8-oxo-dG lesions in pUC19 treated with 40 and 60 μM of Cu_2_TPNap for 4 h at 37°C and compared directly to Cu-Phen and Cu-Terph (reported elsewhere, (44)) and (**C**) M13mp18 single stranded plasmid DNA incubated with increasing concentrations of Cu_2_TPNap for 30 min at 37°C in the absence of added oxidant or reductant.

To identify ROS species involved in the DNA damage process, AMN activity by **Cu_2_TPNap** was investigated (again in the absence of added reductant) using a variety of free radical scavengers: DMSO (hydroxyl radical); tiron (superoxide); pyruvate (hydrogen peroxide); and sodium azide (singlet oxygen). Results show the oxidation mechanism is dependent on superoxide (O_2_^•−^) production as DNA damage was completely inhibited in the presence of tiron (Figure 8A, lanes 10–13). Hydrogen peroxide (H_2_O_2_) is also involved in the cleavage process as pyruvate substantially impeded DNA oxidation (Figure 8A, lanes 14–17). In contrast, the hydroxyl radical (^•^OH) and singlet oxygen (^1^O_2_) were involved to a minor extent only in the **Cu_2_TPNap** DNA degradation pathway (Figure 8A, lanes 6–9 and lanes 18–21). Evidence here suggests that DNA damage is mediated through a superoxide dismutase (SOD) type pathway(68) without significant contributions from competing Fenton or singlet oxygen reactions that produce diffusible ^•^OH and ^1^O_2_ radicals:


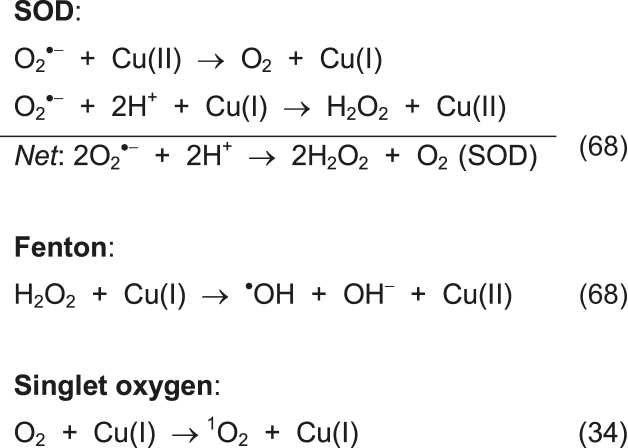


To shed further light on the DNA oxidation pathway, an enzyme-linked immunosorbent assay (ELISA) for the quantitation of 8-oxo-dG lesions was employed. 8-oxo-dG is an important marker for identifying diffusible hydroxyl radicals, which are known to preferentially attack guanosine (2,69) and previous studies have established correlation between DNA oxidation by copper *bis*-1,10-phenanthroline complexes and 8-oxo-dG formation (44,70). In this study, pUC19 DNA exposed to **Cu_2_TPNap** in the presence of added reductant (used to accelerate DNA oxidation, *cf*. Supplementary S-2) produced negligible 8-oxo-dG lesions (<13 nM) whereas >100 nM of 8-oxo-dG was generated by lower concentrations of **Cu-Phen** and **Cu-Terph** (Figure 8B). Further analysis showed the metal chelating agent EDTA to inhibit DNA damage by **Cu_2_TPNap**, while the presence of the copper(I) chelator neocuproine—known to impede **Cu-Phen** mediated DNA damage—had no inhibitory effects (Supplementary S-2). Finally, and in an effort to delineate **Cu_2_TPNap**-mediated DNA damage activity from **Cu-Phen** systems further, the nuclease activity towards single stranded DNA was examined. Here, **Cu_2_TPNap** (40 μM) was found to completely degrade single stranded M13mp18 plasmid DNA in the absence of exogenous reductant over a 30 min window (Figure 8C). Overall, these results contrast with copper phenanthroline systems that are reported to oxidize duplex DNA in the minor groove only (68).

## CONCLUSION

The development of synthetic chemical nucleases is an important research avenue for discovering new DNA-damaging drugs and uncovering new tools for genome editing. Several chemical nucleases, particularly those containing Zn^2+^ and Cu^2+^ cations, have employed pyridyl ligands to anchor and orient metal ions toward nucleic acid substrates to promote efficient cleavage (31–33,71). For example, Williams and co-workers developed a *di*-Zn^2+^*tetra*-2-aminopyridine complex (***di*-Zn-AmPy_4_**) where the metal centres were stabilized by a short propan-2-ol bridge (28). This complex showed efficient Lewis acid activation and phosphodiester hydrolysis of 2-hydroxypropyl 4-nitrophenyl phosphate (HPNPP)—a model system for RNA-like phosphodiesters. *Tetra*-2-pyridine was also used in several DNA targeted *di*-Cu^2+^ complexes with aminobenzyl (72) or aminomethyl–naphthalene (73) bridges. The DNA binding properties of these complexes suggests partial intercalation or unwinding effects and although effective oxidative DNA damaging properties were observed, cleavage did not occur in the absence of exogenous reagents.

This study reports a new *di*-Cu^2+^ metallonuclease, [Cu_2_(*tetra*-(2-pyridyl)-NMe-naphthalene)Cl_4_] (**Cu_2_TPNap**), which incorporates a poly-pyridine ligand scaffold. The complex contains a rigid aromatic naphthalene linker to facilitate *di*-Cu^2+^ interstrand phosphate interactions—enabled by tortional flexibility about the aminomethyl (NMe) bonds—across the major groove of duplex DNA. The agent is a high affinity DNA binder and appears to be incorporated, non-intercalatively, in the major groove of duplex DNA with circular dichroism analysis supporting a major groove binding profile analogous to the classical major groove binder methyl green (MG). Indeed, MG contains three phenyl rings arranged in a propeller-like conformation and is excluded from binding the poly(dA)•2poly(dT) triplex, unlike cationic minor groove binders DAPI, Hoechst 33258 and netropsin that primarily interact with the walls and floor of the narrow minor groove (74). From a structural perspective, therefore, MG and **Cu_2_TPNap** both contain non-coplanar aromatic conformations that render classical intercalation difficult to conceive (74,75).

Direct comparisons between **Cu_2_TPNap** and netropsin in the melphalan protection assay found no minor groove-complex binding interactions and this result, combined with CD analysis and a lack of inhibition to mithramycin A binding in poly[d(G-C)_2_], suggests metal complex residency in the major groove. The binding of metal complexes exclusively in the major groove is rare with the exception of *di*-iron(II) supramolecular helicates developed by Hannon and co-workers (76,77). These helicates, of general formula [Fe_2_L_3_]Cl_4_, bind the major groove of B-DNA and stabilize three-way DNA junctions due to their matching optimum conformation and high cationic (4+) charge (78). Nevertheless, **Cu_2_TPNap** appears to have distinct major groove binding properties that were corroborated by molecular docking studies with the 8-mer d(GGGGCCCC)_2_ (PDB: 2ANA). In these studies the complex was selectively incorporated in the major groove with one Cu(II) ion coordinating a phosphate oxygen atom (Cu•••O(P)) at the second GpG step. A combination of molecular mechanics methods and DFT calculations then revealed the 8-mer d(GGGGCCCC)_2_ fragment to collapse about the complex and this interaction is supported by UV melting experiments with poly[d(G-C)_2_].

**Cu_2_TPNap** is an efficient chemical nuclease capable of producing single strand breaks in the absence of added reductant. While the complex is capable of hydrolysing the model phosphodiester substrate *bis*-(2,4-nitrophenyl)-phosphate (BDNPP), oxidative DNA damage was identified in plasmid pUC19 DNA by the application of T4 DNA ligase. Free radical trapping species then implicated superoxide and hydrogen peroxide as mediators of oxidative damage with the involvement of diffusible free radicals (such as the hydroxyl radical or singlet oxygen) precluded since negligible 8-oxo-dG lesions were detected. In this regard, DNA oxidation by **Cu_2_TPNap** is similar to several *di*- and *tri*-Cu^2+^ state-of-the-art systems reported by Karlin and Rokita (32,79) that differ from copper phenanthroline systems where 8-oxo-dG lesions are produced (7,44,80). Overall, this work represents an important advancement toward the development of new artificial chemical nucleases that exclusively target the major groove of duplex DNA.

## Supplementary Material

Supplementary Data

## References

[1] Cadet J., Douki T., Ravanat J.L. Oxidatively generated base damage to cellular DNA. Free Rad. Biol. Med. 2010; 49:9–21. 20363317 10.1016/j.freeradbiomed.2010.03.025

[2] Gimisis T., Chatgilialoglu C. Oxidatively formed sugar radicals in nucleic acids. Encyclopedia of Radicals in Chemistry, Biology and Materials. 2012; John Wiley & Sons, Ltd 1–26.

[3] Pitié M., Pratviel G. Activation of DNA carbon-hydrogen bonds by metal complexes. Chem. Rev. 2010; 110:1018–1059. 20099805 10.1021/cr900247m

[4] Carell T. DNA Repair. Angew. Chem. Int. Ed. 2015; 54:15330–15333. 10.1002/anie.201509770 26582712

[5] Chen C.B., Mazumder A., Constant J.-F., Sigman D.S. Nuclease activity of 1,10-phenanthroline-copper. New conjugates with low molecular weight targeting ligands. Bioconjug. Chem. 1993; 4:69–77. 7679292 10.1021/bc00019a010

[6] Mancin F., Scrimin P., Tecilla P., Tonellato U. Artificial metallonucleases. Chem. Commun. 2005; 0:2540–2548. 10.1039/b418164f 15900321

[7] Bales B.C., Kodama T., Weledji Y.N., Pitié M., Meunier B., Greenberg M.M. Mechanistic studies on DNA damage by minor groove binding copper–phenanthroline conjugates. Nucleic Acids Res. 2005; 33:5371–5379. 16186134 10.1093/nar/gki856 PMC1235636

[8] Desbouis D., Troitsky I.P., Belousoff M.J., Spiccia L., Graham B. Copper(II), zinc(II) and nickel(II) complexes as nuclease mimetics. Coord. Chem. Rev. 2012; 256:897–937.

[9] Erxleben A. Interactions of copper complexes with nucleic acids. Coord. Chem. Rev. 2018; 360:92–121.

[10] Chen J., Stubbe J. Bleomycins: towards better therapeutics. Nat. Rev. Cancer. 2005; 5:102–112. 15685195 10.1038/nrc1547

[11] Stubbe J., Kozarich J.W. Mechanisms of Bleomycin-Induced DNA degradation. Chem. Rev. 1987; 87:1107–1136.

[12] Sigman D.S., Graham D.R., D’Aurora V., Stern A.M. Oxygen-dependent cleavage of DNA by the 1,10-phenathroline cuporous complex. Inhibition of Escherichia coli DNA polymerase I. J. Biol. Chem. 1979; 254:12269–12272. 387784

[13] Sigman D.S. Chemical Nucleases. Biochemistry. 1990; 29:9097–9105. 2176843 10.1021/bi00491a001

[14] Pitié M., Donnadieu B., Meunier B. Preparation of the new bis(phenanthroline) ligand ‘Clip-Phen’ and evaluation of the nuclease activity of the corresponding copper complex. Inorg. Chem. 1998; 37:3486–3489. 11670431 10.1021/ic980044x

[15] Molphy Z., Prisecaru A., Slator C., Barron N., McCann M., Colleran J., Chandran D., Gathergood N., Kellett A. Copper phenanthrene oxidative chemical nucleases. Inorg. Chem. 2014; 53:5392–5404. 24806421 10.1021/ic500914j

[16] Trejo-Solís C., Palencia G., Zuñiga S., Rodríguez-Ropon A., Osorio-Rico L., Torres Luvia S., Gracia-Mora I., Marquez-Rosado L., Sánchez A., Moreno-García M.E. Cas Ilgly induces apoptosis in glioma C6 cells in vitro and in vivo through Caspase-Dependent and Caspase-Independent mechanisms. Neoplasia. 2005; 7:563–574. 16036107 10.1593/neo.04607 PMC1501287

[17] Galindo-Murillo R., Ruíz-Azuara L., Moreno-Esparza R., Cortés-Guzmán F. Molecular recognition between DNA and a copper-based anticancer complex. Phys. Chem. Chem. Phys. 2012; 14:15539–15546. 23073078 10.1039/c2cp42185b

[18] Rivero-Müller A., De Vizcaya-Ruiz A., Plant N., Ruiz L., Dobrota M. Mixed chelate copper complex, Casiopeina IIgly, binds and degrades nucleic acids: A mechanism of cytotoxicity. Chemico-Biol. Interact. 2007; 165:189–199. 10.1016/j.cbi.2006.12.002 17217939

[19] Hirohama T., Kuranuki Y., Ebina E., Sugizaki T., Arii H., Chikira M., Tamil Selvi P., Palaniandavar M. Copper(II) complexes of 1,10-phenanthroline-derived ligands: studies on DNA binding properties and nuclease activity. J. Inorg. Biochem. 2005; 99:1205–1219. 15833344 10.1016/j.jinorgbio.2005.02.020

[20] Sigman D.S., Mazumder A., Perrin D.M. Chemical Nucleases. Chem. Rev. 1993; 93:2295–2316.

[21] Chen T., Greenberg M.M. Model studies indicate that copper phenanthroline induces direct strand breaks via β-Elimination of the 2′-Deoxyribonolactone intermediate observed in enediyne mediated DNA damage. J. Am. Chem. Soc. 1998; 120:3815–3816.

[22] Bales B.C., Pitié M., Meunier B., Greenberg M.M. A minor groove binding Copper-Phenanthroline conjugate produces direct strand breaks via β-Elimination of 2-Deoxyribonolactone. J. Am. Chem. Soc. 2002; 124:9062–9063. 12149005 10.1021/ja026970z

[23] Galindo-Murillo R., Hernandez-Lima J., González-Rendón M., Cortés-Guzmán F., Ruíz-Azuara L., Moreno-Esparza R. π-Stacking between Casiopeinas® and DNA bases. Phys. Chem. Chem. Phys. 2011; 13:14510–14515. 21748173 10.1039/c1cp20183b

[24] Galindo-Murillo R., García-Ramos J.C., Ruiz-Azuara L., Cheatham T.E., Cortés-Guzmán F. Intercalation processes of copper complexes in DNA. Nucleic Acids Res. 2015; 43:5364–5376. 25958394 10.1093/nar/gkv467 PMC4477671

[25] Serment-Guerrero J., Bravo-Gomez M.E., Lara-Rivera E., Ruiz-Azuara L. Genotoxic assessment of the copper chelated compounds Casiopeinas: Clues about their mechanisms of action. J. Inorg. Biochem. 2017; 166:68–75. 27838580 10.1016/j.jinorgbio.2016.11.007

[26] Yu Z., Han M., Cowan J.A. Toward the design of a catalytic metallodrug: Selective cleavage of G-Quadruplex telomeric DNA by an anticancer Copper-Acridine-ATCUN complex. Angew. Chem. Int. Ed. 2015; 54:1901–1905. 10.1002/anie.201410434 PMC4522906 25504651

[27] Xu Y., Suzuki Y., Lönnberg T., Komiyama M. Human telomeric DNA sequence-specific cleaving by G-quadruplex formation. J. Am. Chem. Soc. 2009; 131:2871–2874. 19209856 10.1021/ja807313x

[28] Feng G., Natale D., Prabaharan R., Mareque-Rivas J.C., Williams N.H. Efficient phosphodiester binding and cleavage by a ZnII complex combining hydrogen-bonding interactions and double lewis acid activation. Angew. Chem. Int. Ed. 2006; 45:7056–7059. 10.1002/anie.200602532 17009384

[29] Humphreys K.J., Karlin K.D., Rokita S.E. Recognition and strand scission at junctions between Single- and Double-Stranded DNA by a trinuclear copper complex. J. Am. Chem. Soc. 2001; 123:5588–5589. 11389647 10.1021/ja010403e

[30] Humphreys K.J., Karlin K.D., Rokita S.E. Targeted strand scission of DNA substrates by a Tricopper(II) coordination complex. J. Am. Chem. Soc. 2002; 124:8055–8066. 12095349 10.1021/ja012539i

[31] Humphreys K.J., Karlin K.D., Rokita S.E. Efficient and specific strand scission of DNA by a dinuclear copper complex: comparative reactivity of complexes with linked Tris(2-pyridylmethyl) amine moieties. J. Am. Chem. Soc. 2002; 124:6009–6019. 12022834 10.1021/ja020039z

[32] Li L., Karlin K.D., Rokita S.E. Changing selectivity of DNA oxidation from deoxyribose to guanine by ligand design and a new binuclear copper complex. J. Am. Chem. Soc. 2005; 127:520–521. 15643865 10.1021/ja044209e

[33] Thyagarajan S., Murthy N.N., Narducci Sarjeant A.A., Karlin K.D., Rokita S.E. Selective DNA strand scission with binuclear copper complexes: Implications for an active Cu2 −O2 species. J. Am. Chem. Soc. 2006; 128:7003–7008. 16719480 10.1021/ja061014t PMC2556057

[34] Slator C., Molphy Z., McKee V., Long C., Brown T., Kellett A. Di-copper metallodrugs promote NCI-60 chemotherapy via singlet oxygen and superoxide production with tandem TA/TA and AT/AT oligonucleotide discrimination. Nucleic Acids Res. 2018; 46:2733–2750. 29474633 10.1093/nar/gky105 PMC5888725

[35] Hamann J.N., Rolff M., Tuczek F. Monooxygenation of an appended phenol in a model system of tyrosinase: implications on the enzymatic reaction mechanism. Dalton Trans. 2015; 44:3251–3258. 25597816 10.1039/c4dt03010a

[36] McCann M., McGinley J., Ni K., O’Connor M., Kavanagh K., McKee V., Colleran J., Devereux M., Gathergood N., Barron N. A new phenanthroline-oxazine ligand: synthesis, coordination chemistry and atypical DNA binding interaction. Chem. Commun. 2013; 49:2341–2343. 10.1039/c3cc38710k 23407675

[37] Prisecaru A., Molphy Z., Kipping R.G., Peterson E.J., Qu Y., Kellett A., Farrell N.P. The phosphate clamp: sequence selective nucleic acid binding profiles and conformational induction of endonuclease inhibition by cationic Triplatin complexes. Nucleic Acids Res. 2014; 42:13474–13487. 25414347 10.1093/nar/gku1157 PMC4267626

[38] Coleman F., Hynes M.J., Erxleben A. GaIII complexes as models for the MIII site of purple acid phosphatase: Ligand effects on the hydrolytic reactivity toward bis(2,4-dinitrophenyl) phosphate. Inorg. Chem. 2010; 49:6725–6733. 20565083 10.1021/ic100722w

[39] Martel A.E., Smith R.M. Critical Stability Constants. 1989; Boston Springer.

[40] Bunton C.A., Farber S.J. Acid catalyzed hydrolyses of weakly basic phosphate esters. J. Org. Chem. 1969; 34:3396–3403.

[41] Prisecaru A., Devereux M., Barron N., McCann M., Colleran J., Casey A., McKee V., Kellett A. Potent oxidative DNA cleavage by the di-copper cytotoxin: [Cu_2_([μ-terephthalate)(1,10-phen)_4_]^2+^. Chem. Commun. 2012; 48:6906–6908. 10.1039/c2cc31023f 22673761

[42] Wang P., Bauer G.B., Bennett R.A.O., Povirk L.F. Thermolabile adenine adducts and A-T base pair substitutions induced by nitrogen mustard analogues in an SV40-based shuttle plasmid. Biochemistry. 1991; 30:11515–11521. 1660721 10.1021/bi00113a005

[43] Qu Y., Moniodis J.J., Harris A.L., Yang X., Hegmans A., Povirk L.F., Berners-Price S.J., Farrell N.P. Non-Covalent polynuclear platinum compounds as polyamine analogs. RSC Drug Discovery Series No. 17 Polyamine Drug Discovery. 2011; 191–204.

[44] Molphy Z., Slator C., Chatgilialoglu C., Kellett A. DNA oxidation profiles of copper phenanthrene chemical nucleases. Front. Chem. 2015; 3:1–9. 25954741 10.3389/fchem.2015.00028 PMC4404973

[45] Morris G.M., Huey R., Lindstrom W., Sanner M.F., Belew R.K., Goodsell D.S., Olson A.J. AutoDock4 and AutoDockTools4: automated docking with selective receptor flexibility. J. Comput. Chem. 2009; 30:2785–2791. 19399780 10.1002/jcc.21256 PMC2760638

[46] McCall M., Brown T., Kennard O. The crystal structure of d(G-G-G-G-C-C-C-C): a model for Poly(dG)Poly(dG). J. Mol. Biol. 1985; 183:385–396. 4020865 10.1016/0022-2836(85)90009-9

[47] Rappé A.K., Casewit C.J., Colwell K.S., Goddard W.A., Skiff W.M. UFF, a full periodic table force field for molecular mechanics and molecular dynamics simulations. J. Am. Chem. Soc. 1992; 114:10024–10035.

[48] Rappé A.K., Goddard W.A. Charge equilibration for molecular dynamics simulations. J. Phys. Chem. 1991; 95:3358–3363.

[49] Maseras F., Morokuma K. IMOMM: A new integrated ab initio + molecular mechanics geometry optimization scheme of equilibrium structures and transition states. J. Comput. Chem. 1995; 16:1170–1179.

[50] Humbel S., Sieber S., Morokuma K. The IMOMO method: Integration of different levels of molecular orbital approximations for geometry optimization of large systems: Test for n‐butane conformation and SN2 reaction: RCl+Cl−. J. Chem. Phys. 1996; 105:1959–1967.

[51] Matsubara T., Sieber S., Morokuma K. A test of the new “integrated MO + MM” (IMOMM) method for the conformational energy of ethane and n‐butane. Int. J. Quantum Chem. 1996; 60:1101–1109.

[52] Svensson M., Humbel S., Froese R.D.J., Matsubara T., Sieber S., Morokuma K. ONIOM: A multilayered integrated MO + MM method for geometry optimizations and single point energy predictions. A test for Diels−Alder reactions and Pt(P(t-Bu)_3_)_2_ + H_2_ oxidative addition. J. Phys. Chem. 1996; 100:19357–19363.

[53] Svensson M., Humbel S., Morokuma K. Energetics using the single point IMOMO (integrated molecular orbital+molecular orbital) calculations: Choices of computational levels and model system. J. Chem. Phys. 1996; 105:3654–3661.

[54] Vreven T., Morokuma K. On the application of the IMOMO (integrated molecular orbital + molecular orbital) method. J. Comput. Chem. 2000; 21:1419–1432.

[55] Becke A.D. Density‐functional thermochemistry. III. The role of exact exchange. J. Chem. Phys. 1993; 98:5648–5652.

[56] Becke A.D. Density-functional exchange-energy approximation with correct asymptotic behavior. Phys. Rev. A. 1988; 38:3098–3100. 10.1103/physreva.38.3098 9900728

[57] Dunning T.H., Hay P.J. Schaefer HF Gaussian basis sets for molecular calculations. Methods of Electronic Structure Theory. Modern Theoretical Chemistry. 1977; 3:NY Plenum Press 1–28.

[58] Pieper R.O., Erickson L.C. DNA adenine adducts induced by nitrogen mustards and their role in transcription termination in vitro. Carcinogenesis. 1990; 11:1739–1746. 2208589 10.1093/carcin/11.10.1739

[59] Bauer G.B., Povirk L.F. Specificity and kinetics of interstrand and intrastrand bifunctional alkylation by nitrogen mustards at a G-G-C sequence. Nucleic Acids Res. 1997; 25:1211–1218. 9092631 10.1093/nar/25.6.1211 PMC146567

[60] Lombó F., Menéndez N., Salas J.A., Méndez C. The aureolic acid family of antitumor compounds: structure, mode of action, biosynthesis, and novel derivatives. Appl. Microbiol. Biotechnol. 2006; 73:1–14. 17013601 10.1007/s00253-006-0511-6

[61] Van Dyke M.W., Dervan P.B. Chromomycin, mithramycin, and olivomycin binding sites on heterogeneous DNA. Footprinting with methidiumpropyl-EDTA.cntdot.iron(II). Biochemistry. 1983; 22:2373–2377. 6222762 10.1021/bi00279a011

[62] Fox K.R., Howarth N.R. Investigations into the sequence-selective binding of mithramycin and related ligands to DNA. Nucleic Acids Res. 1985; 13:8695–8714. 2934687 10.1093/nar/13.24.8695 PMC318945

[63] Keniry M.A., Banville D.L., Simmonds P.M., Shafer R. Nuclear magnetic resonance comparison of the binding sites of mithramycin and chromomycin on the Self-complementary oligonucleotide d(ACCCGGGT)2: evidence that the saccharide chains have a role in sequence specificity. J. Mol. Biol. 1993; 231:753–767. 8515449 10.1006/jmbi.1993.1324

[64] Sastry M., Fiala R., Patel D. Solution structure of mithramycin dimers bound to partially overlapping sites on DNA. J. Mol. Biol. 1995; 251:674–689. 7666419 10.1006/jmbi.1995.0464

[65] Barceló F., Scotta C., Ortiz-Lombardía M., Méndez C., Salas J.A., Portugal J. Entropically-driven binding of mithramycin in the minor groove of C/G-rich DNA sequences. Nucleic Acids Res. 2007; 35:2215–2226. 17369273 10.1093/nar/gkm037 PMC1874653

[66] Povirk L.F., Wübter W., Köhnlein W., Hutchinson F. DNA double-strand breaks and alkali-labile bonds produced by bleomycin. Nucleic Acids Res. 1977; 4:3573–3580. 73164 10.1093/nar/4.10.3573 PMC342673

[67] Ferretti L., Sgaramella V. Specific and reversible inhibition of the blunt end joining activity of the T4 DNA ligase. Nucleic Acids Res. 1981; 9:3695–3705. 6269089 10.1093/nar/9.15.3695 PMC327385

[68] Halliwell B., Gutteridge J. Chapter 6: Measurement of reactive species. Free Radicals in Biology and Medicine. 2015; Oxford University Press.

[69] Terzidis M.A., Prisecaru A., Molphy Z., Barron N., Randazzo A., Dumont E., Krokidis M.G., Kellett A., Chatgilialoglu C. Radical-induced purine lesion formation is dependent on DNA helical topology. Free Rad. Res. 2016; 50:S91–S101. 10.1080/10715762.2016.1244820 27733084

[70] Frelon S., Douki T., Favier A., Cadet J. Hydroxyl radical is not the main reactive species involved in the degradation of DNA bases by copper in the presence of hydrogen peroxide. Chem. Res. Toxicol. 2003; 16:191–197. 12588190 10.1021/tx025650q

[71] Mohamed M.F., Sánchez-Lombardo I., Neverov A.A., Brown R.S. Solvent induced cooperativity of Zn(II) complexes cleaving a phosphatediester RNA analog in methanol. Org. Biomol. Chem. 2012; 10:631–639. 22116167 10.1039/c1ob06482g

[72] Zhao Y., Zhu J., He W., Yang Z., Zhu Y., Li Y., Zhang J., Guo Z. Oxidative DNA cleavage promoted by multinuclear copper complexes: activity dependence on the complex structure. Chem. Eur. J. 2006; 12:6621–6629. 16755636 10.1002/chem.200600044

[73] Sun J., Deng S.-Y., Zhang L., He J., Jiang L., Mao Z.-W., Ji L.-N. DNA affinity and cleavage by naphthalene-based mononuclear and dinuclear copper(II) complexes. J. Coord. Chem. 2009; 62:3284–3295.

[74] Kim S.K., Nordén B. Methyl green. A DNA major-groove binding drug. Fed. Eur. Biochem. Soc. Lett. 1993; 315:61–64. 10.1016/0014-5793(93)81133-k 8416812

[75] Bailly C., Hénichart J.-P., Colson J.-P., Colson P., Houssier C. Drug-DNA sequence-dependent interactions analysed by electric linear dichroism. J. Mol. Recognit. 1992; 5:155–171. 1339484 10.1002/jmr.300050406

[76] Hannon M.J., Moreno V., Prieto M.J., Moldrheim E., Sletten E., Meistermann I., Isaac C.J., Sanders K.J., Rodger A. Intramolecular DNA coiling mediated by a Metallo-Supramolecular cylinder. Angew. Chem. Int. Ed. 2001; 40:879–884. 10.1002/1521-3773(20010302)40:5<879::AID-ANIE879>3.0.CO;2-X 29712178

[77] Malina J., Hannon M.J., Brabec V. Iron(II) supramolecular helicates condense plasmid DNA and inhibit vital DNA-Related enzymatic activities. Chem. Eur. J. 2015; 21:11189–11195. 26103944 10.1002/chem.201501307

[78] Oleksi A., Blanco A.G., Boer R., Usón I., Aymamí J., Rodger A., Hannon M.J., Coll M. Molecular recognition of a Three-Way DNA junction by a Metallosupramolecular Helicate. Angew. Chem. 2006; 118:1249–1253. 10.1002/anie.200503822 16463312

[79] Li L., Murthy N.N., Telser J., Zakharov L.N., Yap G.P.A., Rheingold A.L., Karlin K.D., Rokita S.E. Targeted guanine oxidation by a Dinuclear Copper(II) complex at single Stranded/Double stranded DNA junctions. Inorg. Chem. 2006; 45:7144–7159. 16933915 10.1021/ic0605930

[80] Cadet J., Douki T., Ravanat J.L. Oxidatively generated damage to the guanine moiety of DNA: Mechanistic aspects and formation in cells. Acc. Chem. Res. 2008; 41:1075–1083. 18666785 10.1021/ar700245e

