# Supplementary material for: A phosphate-targeted dinuclear Cu(II) complex combining major groove binding and oxidative DNA cleavage
Source: Nucleic Acids Res. 2018 Sep 17;46(19):9918–31. doi: 10.1093/nar/gky806 (PMC6212767; doi:10.1093/nar/gky806)
Supplement: Supplementary Data [file gky806_supplemental_files.zip › Cu2TPNap_ESI.pdf]

Supporting Information

Accompanying the manuscript

A Phosphate-targeted dinuclear Cu(II) complex combining major groove binding and oxidative DNA cleavage

Zara Molphy,[1] Diego Montagner,[2] Satish S. Bhat,[3] Creina Slator,[1] Conor Long,[1]
Andrea Erxleben*[3,4] and Andrew Kellett*[1,5]

(S-1) X-ray Crystallography

(S-2) DNA Binding and Cleavage Studies

(S-1) X-Ray Crystallography

Single crystals of **Cu_2TPNap** suitable for single crystal X-ray diffraction were grown by slow evaporation of a solution of the complex in CH_3CN at room temperature. Crystal data were collected at room temperature on an Agilent (formerly OxfordDiffraction) Xcalibur CCD diffractometer using graphite-monochromated Mo-K_{α} radiation (λ= 0.71069 Å).(1) The structure was solved by direct methods and subsequent Fourier syntheses and refined by full-matrix least squares on F^2 using SHELXS-97 and SHELXL-97 within the Oscail package).(2, 3) The scattering factors were those given in the SHELXL program. Hydrogen atoms were generated geometrically and refined as riding atoms with isotropic displacement factors equivalent to 1.2 times those of the atom to which they were attached. Graphics were produced with ORTEX.(4) This structure has been deposited with the Cambridge Crystallographic Data Centre (CCDC 1407620).

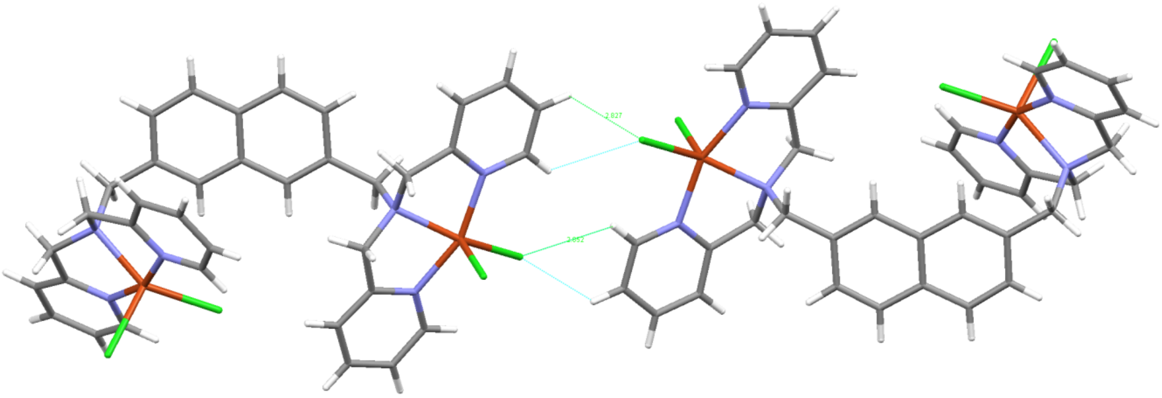

Figure S-1. Packing diagram of **Cu_2TPNap·H_2O** showing inter-molecular hydrogen bonding interactions. For the sake of clarity, water molecules of crystallization are not shown.

Crystallographic data are given in Table **S-1**, and a list of selected bond lengths and angles can be found in Table **S-2**.

Table S-1. Crystal data and structure refinement parameters for **Cu_2TPNap**.

Chemical formula	$C_{36}H_{36}Cl_4Cu_2N_6O$
Formula weight	837.59
Crystal system, space group	Triclinic, *P*-1
Temperature (K)	293
Unit Cell Dimensions	a=9.5092 (3) Å, α=83.693 (3)°<br>b=14.282 (6) Å, β=71.530 (3)°<br>c=14.982 (5) Å, γ=74.327 (3)°
V (Å^3)	1857.62 (12)
Z	2
F(000)	856
Density (Mg m^{-3})	1.497
No. of reflections for cell measurement	7595
ɵ range (°) for cell measurement	3.0–26.4
μ (mm^{-1})	1.47
Crystal shape	Prism
Colour	Blue
Crystal size (mm)	0.40 × 0.35 × 0.20
No. of measured, independent and observed [$I > 2\sigma(I)$] reflections	14030, 7595, 5992
θ values (°)	θ_{max} = 26.4, θ_{min} = 3.0
$(\sin \theta/\lambda)_{max}$ (Å^{-1})	0.625
No. of reflections	7595
No. of parameters	442
No. of restraints	0
R_{int}	0.026
$R[F^2 > 2\sigma(F^2)]$, $wR(F^2)$, S	0.037, 0.102, 1.05

Table S-2. Selected bond lengths [Å] and angles [°] for complex **Cu_2TPNap**.

**Bond lengths**			
Cu1—N3	2.000 (2)	Cu2—N6	2.006 (2)
Cu1—N2	2.001 (2)	Cu2—N5	2.023 (2)
Cu1—N1	2.086 (2)	Cu2—N4	2.078 (2)
Cu1—Cl2	2.3020 (7)	Cu2—Cl4	2.2496 (8)
Cu1—Cl1	2.4632 (9)	Cu2—Cl3	2.4483 (8)
**Bond angles**			
N3—Cu1—N1	81.77 (9)	N5—Cu2—N4	80.61 (9)
N2—Cu1—N1	80.73 (9)	N6—Cu2—Cl4	97.08 (7)
N3—Cu1—Cl2	98.09 (7)	N5—Cu2—Cl4	97.54 (7)
N2—Cu1—Cl2	96.64 (7)	N4—Cu2—Cl4	154.34 (7)
N3—Cu1—Cl1	91.55 (7)	N6—Cu2—Cl3	96.10 (8)
N2—Cu1—Cl1	93.53 (7)	N5—Cu2—Cl3	93.44 (7)
N1—Cu1—Cl1	109.07 (7)	N4—Cu2—Cl3	99.95 (7)
Cl2—Cu1—Cl1	105.72 (3)	Cl4—Cu2—Cl3	105.71 (3)
N6—Cu2—N4	80.10 (9)		

Table S-3. Photophysical properties of complex **Cu_2TPNap** at room temperature.

**Absorption, λ_{max} (nm), (ε ($M^{-1}cm^{-1}$)**<br>**Ligand Transitions (MLCT)**	**Emission**<br>**λ_{em} (nm)**
232 (31658), 260 (17174), 676 (209)<br>294 (4672)	370

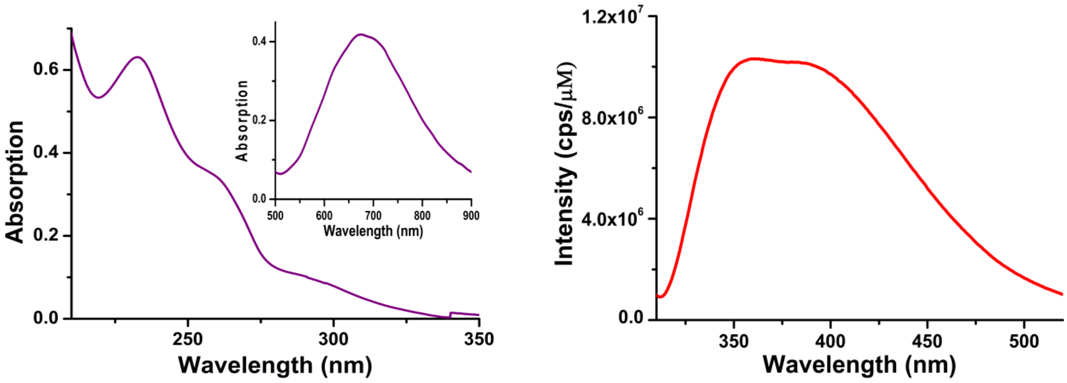

Figure S-2. Absorption **(A)** and emission **(B)** spectra of complex **Cu_2TPNap** (20 μM) in methanol at room temperature. (**(A)** Inset corresponds to absorption spectra of **Cu_2TPNap** in visible region at 2 mM concentration).

(S-2) DNA Binding Studies

DNA fluorescence quenching

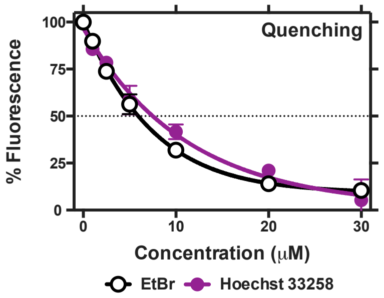

Figure S-3. DNA fluorescence quenching of limited EtBr (5 μM) or Hoechst 33258 (5 μM) bound ctDNA (25 μM) in the presence of increasing concentrations of **Cu_2TPNap**.(5)

Thermal Melting

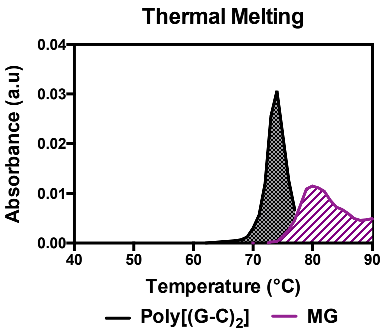

Figure S-4. Thermal melting of Poly[d(G-C)$_2$] only and Poly[d(G-C)$_2$] treated with r = 0.1 loading of major groove binging agent methyl green.

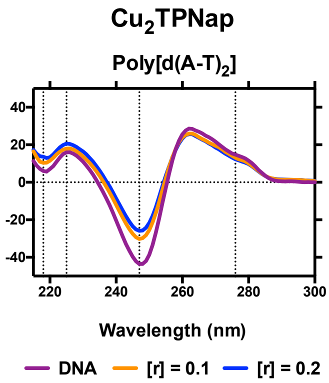

Figure S-5. Full CD spectral profile of salmon testes (stDNA) and alternating co-polymers poly[d(A-T)$_2$] and poly[d(G-C$_2$)] in the presence of **Cu_2TPNap** at r =0.1 and r =0.2 loading.

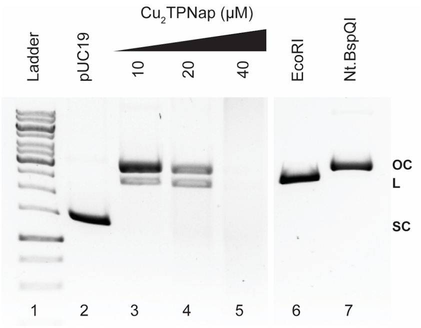

Figure S-6. DNA cleavage reactions by **Cu_2TPNap** on pUC19 over 5 hours at 37 °C in the absence of added exogenous reductant.

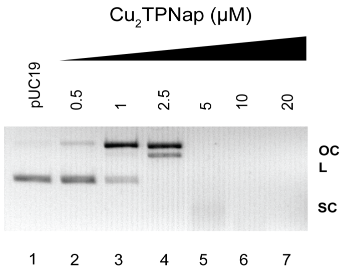

Figure S-7. Cleavage of 400 ng pUC19 plasmid DNA by increasing concentrations of **Cu_2TPNap** in the presence of 25 mM NaCl and 1mM Na-*L*-ascorbate over 30 min at 37 °C.

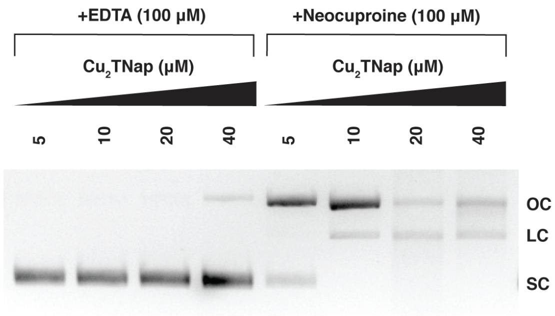

Figure S-8. Cu_2TPNap pUC19 DNA cleavage experiments in the presence of copper chelating agents ethylenediaminetetraacetic acid (EDTA) and neocuproine and 25 mM NaCl over a 1 hour exposure period at 37 °C.

REFERENCES

1. CrysAlisPro, Oxford Diffraction Ltd., Version 1.171.33.31 (release 08-01-2009 CrysAlis171.NET)

2. Sheldrick,G.M. (2015) SHELXT - Integrated space-group and crystal-structure determination. *Acta Crystallogr. Sect. A Found. Crystallogr.*, **71**, 3–8.

3. Sheldrick,G.M. (2015) Crystal structure refinement with SHELXL. *Acta Crystallogr. Sect. C Struct. Chem.*, **71**, 3–8.

4. McArdle,P., Gilligan,K., Cunningham,D., Dark,R. and Mahon,M. (2004) A method for the prediction of the crystal structure of ionic organic compounds - the crystal structures of o-toluidinium chloride and bromide and polymorphism of bicifadine hydrochloride. *CrystEngComm*, **6**, 303–309.

5. McCann,M., McGinley,J., Ni,K., O'Connor,M., Kavanagh,K., McKee,V., Colleran,J., Devereux,M., Gathergood,N., Barron,N., et al. (2013) A new phenanthroline-oxazine ligand: synthesis, coordination chemistry and atypical DNA binding interaction. *Chem. Commun.*, **49**, 2341–2343.
